# Next-Generation Wound Care: A Scoping Review on Probiotic, Prebiotic, Synbiotic, and Postbiotic Cutaneous Formulations

**DOI:** 10.3390/ph18050704

**Published:** 2025-05-09

**Authors:** Patrícia Machado, Felipe Neme Ribeiro, Fernanda Cristina Wroblevski Giublin, Naomi Gerzvolf Mieres, Fernanda Stumpf Tonin, Roberto Pontarolo, Marcel Henrique Marcondes Sari, Raul Edison Luna Lazo, Luana Mota Ferreira

**Affiliations:** 1 Programa de Pós-Graduação em Ciências Farmacêuticas, Universidade Federal do Paraná, Curitiba 80210-170, Brazil; patricia.machado.ufpr@gmail.com (P.M.); nmgerzvolf@gmail.com (N.G.M.); pontarolo@ufpr.br (R.P.); marcelsari@ufpr.br (M.H.M.S.); 2 Departamento de Medicina, Universidade Federal do Paraná, Curitiba 80210-170, Brazil; felipeneme@ufpr.br; 3 Complexo do Hospital de Clínicas, Ambulatório de Dermatologia, Universidade Federal do Paraná, Curitiba 80060-900, Brazil; wroblevski@gmail.com; 4 Health and Technology Research Center (H&TRC), Escola Superior de Tecnologia da Saúde (ESTeSL-IPL), 1990-096 Lisbon, Portugal; fer_stumpf_tonin@hotmail.com; 5 Pharmacy and Pharmaceutical Technology Department, Social and Legal Pharmacy Section, University of Granada, 18012 Granada, Spain

**Keywords:** cutaneous administration, dermatological formulations, burned skin, excisional skin, scar formation, tissue repair

## Abstract

**Background/Objectives**: Chronic wounds represent a significant socioeconomic burden, affecting 1–2% of the global population. Wound healing is a complex process involving inflammation, cell proliferation, and tissue remodeling, but factors such as infections, diabetes, aging, and poor nutrition can impair recovery, leading to chronic wounds. Given these challenges, researchers have explored topical probiotics, synbiotics, and postbiotics as alternatives strategies. Strains like *Lactobacillus* and *Bifidobacterium* contribute to skin restoration by producing antimicrobial, anti-inflammatory, and immunomodulatory compounds, offering a novel approach to cutaneous restoration. Our study aims to address the potential effects of topical probiotic, synbiotic, and postbiotic formulations for wound healing applications by means of a broad scoping review and evidence-gap mapping. **Methods**: A systematic literature search of preclinical studies (in vitro and in vivo) was performed in PubMed, Scopus, and Web of Science (January 2025), yielding 3052 articles after duplicates removal, of which 44 met the inclusion criteria. **Results**: These studies were published between 1986 and 2024, mostly by China (27.3%) and Iran (25.0%). Probiotics were frequently evaluated among the studies included (47.7%) (with *Lactobacillus plantarum* being the most assessed strain), followed by postbiotics (36.4%) (with predominant use of cell-free supernatants) and synbiotics (15.9%) (especially fructooligosaccharides). Dosage forms included gels (44.4%), films (15.6%), and ointments (13.3%). **Conclusions**: Most studies indicate that probiotics, synbiotics, and postbiotics have antimicrobial and anti-inflammatory properties, while promoting angiogenesis, tissue regeneration, and skin barrier restoration. The use of different delivery systems may additionally enhance therapeutic outcomes by accelerating wound closure, reducing bacterial load, and modulating immune response. However, methodological limitations in animal studies highlight the need for greater experimental rigor. Further robust clinical trials are essential to confirm efficacy and safety before clinical application of these formulations.

## 1. Introduction

Chronic wounds, mainly derived from cutaneous and subcutaneous diseases, represent a growing socioeconomic issue in the Western world, with a significant impact on patients’ physical health, mental well-being, and overall quality of life [1]. It is estimated that approximately 1–2% of the global population will experience a chronic wound at some point in their lives [2]. A retrospective analysis from 2018 found that around 8.2 million people had wounds, regardless of infection status. Cost estimates for treating acute and chronic wounds under Medicare ranged between $28.1 billion and $96.8 billion [3]. In Europe, an estimated 1.5 to 2 million people suffer from acute or chronic wounds [4]. Additionally, approximately 15% of patients develop complications due to scar formation following surgery or trauma, as well as burns [5,6].

Chronic wounds arise from various causes, with diabetic foot ulcers, venous leg ulcers, arterial ulcers, and pressure injuries being the most common globally [7,8]. Diabetic foot ulcers are notably prevalent among those with poorly managed diabetes, leading to issues such as impaired microcirculation, neuropathy, and delayed tissue repair [7]. Venous leg ulcers stem from chronic venous insufficiency, while arterial ulcers are linked to peripheral artery disease and tissue ischemia. Pressure injuries are often found in immobile or older patients. Each wound type displays unique pathophysiological characteristics, including differences in microbiota composition, inflammatory responses, and healing processes, all of which may affect the efficacy of topical probiotic and postbiotic treatments [9]. For instance, probiotic therapies that promote angiogenesis and mitigate oxidative stress may be particularly beneficial for diabetic wounds, whereas approaches that focus on reducing inflammation and preventing biofilm formation are vital for treating venous ulcers [10,11]. Additionally, the occurrence of chronic wounds varies significantly by region. Diabetic foot ulcers are more frequent in low- and middle-income nations due to rising diabetes rates and limited access to preventive measures, whereas pressure injuries are increasingly observed in aging populations in high-income countries [3]. These geographical variations influence healthcare systems, necessitating customized management approaches, including creating specialized wound care solutions.

Wound healing is a complex biological process that involves a series of coordinated cellular and molecular events, including inflammation, cell proliferation, and tissue remodeling [12,13]. Although it is a natural repair mechanism of the body, factors such as infections, chronic diseases (diabetes mellitus), advanced age, and poor nutrition can impair this process, leading to the formation of chronic and difficult-to-heal wounds [14,15,16]. This scenario represents a significant challenge for health systems and negatively impacts patients’ quality of life. Chronic systemic conditions like diabetes mellitus and aging not only hinder cellular and vascular responses essential for wound healing but also significantly alter the composition and function of the skin microbiome. In those with diabetes, hyperglycemia induces chronic low-grade inflammation, resulting in dysbiosis marked by reduced beneficial bacterial populations and increased harmful species [11]. This microbial imbalance worsens local inflammatory responses, disrupts keratinocyte function, and delays re-epithelialization. Likewise, aging is linked to immunosenescence and changes in skin barrier function, leading to shifts in the microbiota towards more pro-inflammatory profiles, which diminishes wound healing capacity [9]. These interactions underscore the importance of preserving a balanced skin microbiome for wound repair and support the rationale for developing therapeutic strategies based on probiotics and postbiotics.

Recently, the role of the microbiome in skin health has received increasing attention, with studies highlighting its influence in modulating the healing process. Imbalances in the cutaneous microbiota, often associated with infections by opportunistic pathogens, can delay tissue regeneration and contribute to complications. Studies indicate that alterations in the microbiota can significantly affect wound healing dynamics, once commensal bacteria play a crucial role in modulating immune responses, promoting keratinocyte proliferation, and supporting angiogenesis. These interactions highlight the importance of a balanced microbial community in maintaining skin health and optimizing the healing process following injury [14].

In this context, probiotics, prebiotics, postbiotics, and synbiotics have recently emerged as promising therapeutic approaches. Probiotics and their derivatives, including culture supernatants and mixed probiotics, can accelerate wound healing due to their antibiofilm, immunomodulatory effects [17], antibacterial and anti-inflammatory activities, and angiogenesis characteristics that lead to the promotion of wound healing [18]. Specific probiotic strains have shown particular promise in modulating wound healing processes. *Lactobacillus plantarum* and *Lactobacillus reuteri* have been extensively studied for their antimicrobial, anti-inflammatory, and regenerative properties, including stimulation of fibroblast proliferation and angiogenesis [19,20]. Additionally, *Bifidobacterium bifidum* has demonstrated potential in enhancing fibroblast activity and reducing bacterial burden in experimental models [21]. Despite their proven efficacy in the treatment of wound infections, the intimate mechanisms of probiotics in wound healing are not fully explored [17].

Cutaneous formulations containing probiotics and their derivatives can offer an innovative strategy to promote wound healing, due to their ability to create an environment favorable to tissue regeneration. These microorganisms can modulate the local immune response, compete with pathogens for nutrients and space, and release beneficial metabolites, such as short-chain fatty acids and bacteriocins, which promote skin homeostasis. Different pharmaceutical dosage forms, such as hydrogels, films, and ointments, have been used to protect probiotics [22]. Moreover, various biopolymers and hydrogels are currently being studied for their characteristics as wound dressings. Gels offer hydration, flexibility, and enhanced bioadhesion, making them particularly suitable for irregular or exudative wounds [23,24], while films provide protective barriers and are advantageous for treating larger or superficial wound areas [25,26]. These materials offer unique properties such as biocompatibility, antibacterial effects, and the ability to control microbial attachment, colonization, and biofilm development. Additionally, they are biodegradable, have low cytotoxicity, and can incorporate and enhance the beneficial effects of probiotics and prebiotics on wound healing [17]. However, challenges such as maintaining probiotic viability and ensuring effective delivery to deep wound sites remain.

Although traditional therapeutic approaches for wound healing remain widely used, synthesized evidence on the benefits or otherwise of topical application of probiotic, prebiotic, synbiotic, and postbiotic formulations remains underexplored. This study addresses these knowledge gaps by mapping the available data on using probiotic, synbiotic, and postbiotic formulations for wound healing. Additionally, it seeks to provide a comprehensive overview of the current state of topical probiotic-based formulations and its derivative formulations, highlighting their therapeutic potential in dermatology and proposing novel strategies for managing chronic wounds.

## 2. Results

**Figure 1:**
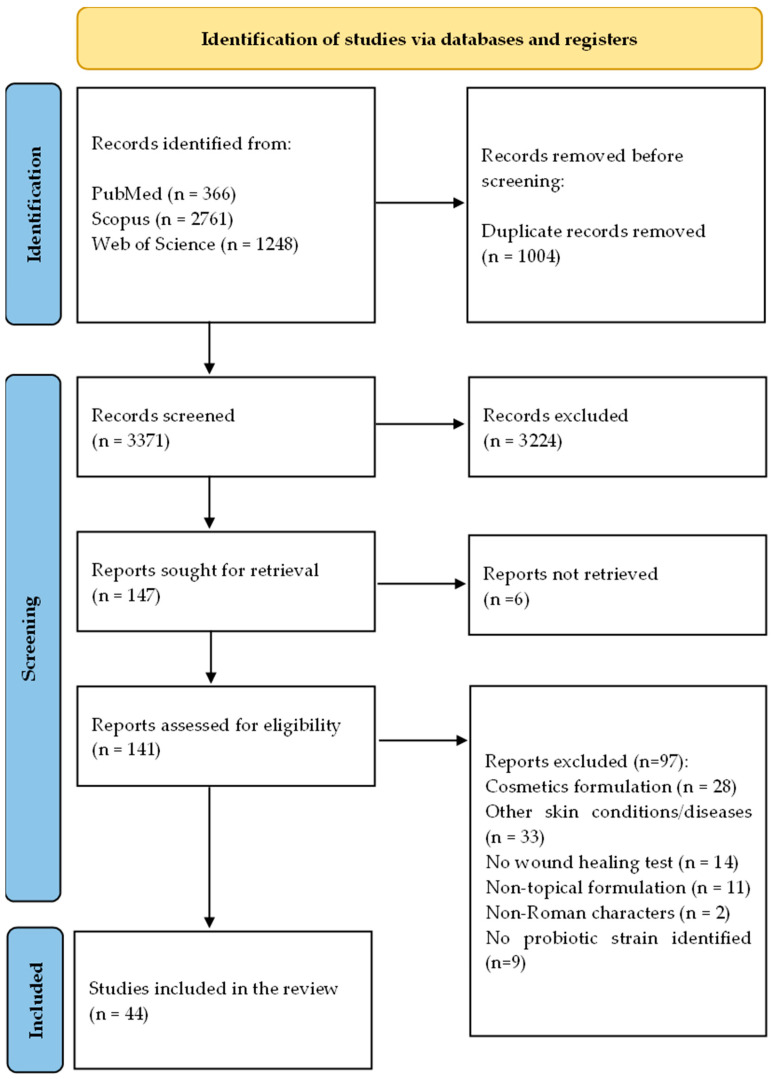
Scoping review flowchart.

A total of 4375 articles were identified from Scopus, Web of Science, and PubMed databases. After removing duplicates, 3052 records were screened by reading the titles and abstracts, of which 3371 articles were considered irrelevant to the research. From the 141 studies read in full-text, 97 were excluded as they did not meet the inclusion criteria, leaving 44 studies for data extraction and synthesis (Figure 1). Excluded studies and reasons are in the Supplementary Materials, Supplementary Table S1.

In terms of geographic distribution, the countries with the highest number of publications were China (27.3%) and Iran (25.0%), followed by India (11.4%) and Germany (4.5%). Other countries, including the USA, Japan, Portugal, Canada, Pakistan, Iraq, Israel, Taiwan, South Korea, Brazil, Italy, Tunisia, Russia, and Kazakhstan, accounted for 2.3% of the total publications. The selected studies were published between 1986 and 2024, with a notable increase in publications in recent years, with 10 (22.7%) published in 2024. The predominance of publications from countries such as China, Iran, and India may reflect regional wound care practices that traditionally integrate natural and alternative therapies and recent investments in regenerative medicine. Additionally, the steady increase in publications observed since 2018 likely corresponds to a broader global trend of exploring topical applications of probiotics and postbiotics, driven by advancements in delivery technologies and growing support for research into alternative strategies for chronic wound management.

### 2.1. Evidence-Gap Mapping

**Figure 2:**
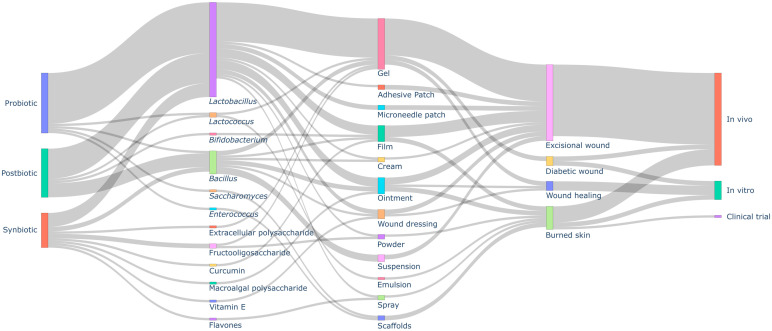
Sankey diagram of the available evidence on cutaneous formulations containing probiotics, postbiotics, and synbiotics for treating wounds. The analysis includes different types of microorganisms, pharmaceutical forms, types of wounds, and study models (in vitro, in vivo, and clinical trials).

Figure 2 shows an overview of probiotic-, synbiotic-, and postbiotic-based formulations as potential therapy for wound healing. Probiotics were the most frequently used microbiome-modulating agents (47.7%, *n* = 21), followed by postbiotics (36.4%, *n* = 16) and synbiotics (15.9%, *n* = 7). Formulations containing only prebiotics were not found.

The most common dosage forms included gels (44.4%, *n* = 20), films (15.6%, *n* = 7), and ointments (13.3%, *n* = 6). Other formulations, such as scaffolds, wound dressings, and microneedle patches (each 4.4%, *n* = 2) were also mentioned. Less frequent forms included suspensions, creams, emulsions, sprays, adhesive patches, and powders (*n* = 1 study each).

The most frequently studied probiotic strain was *Lactobacillus plantarum* (19.7%, *n* = 12), followed by *Lactobacillus reuteri* (11.5%, *n* = 7), *Lactobacillus casei* (6.6%, *n* = 4), and *Lactobacillus fermentum* (6.6%, *n* = 4). Other commonly used strains included *Lactiplantibacillus plantarum*, *Lactobacillus acidophilus*, and *Bacillus subtilis* (each 4.9%, *n* = 3). Less frequently reported strains include *Bacillus amyloliquefaciens*, *Bacillus velezensis*, *Limosilactobacillus fermentum*, *Saccharomyces cerevisiae*, and *Enterococcus mundtii*. See the main results in Table 1. Among the prebiotics in the synbiotic formulations, fructooligosaccharide (FOS) was the most commonly used (28.6%, *n* = 2), followed by curcumin, vitamin E, flavones, extracellular polysaccharides, and macroalgal polysaccharides (each at 14.3%, *n* = 1). See Table 2. For postbiotics, cell-free supernatant was the most frequently studied (68.8%, *n* = 11), followed by cell-free extracellular vesicles (12.5%, *n* = 2). Other postbiotics, including lactosporin, exopolysaccharides, and *Lactococcus lactis* lysate, were also mentioned (6.3%, *n* = 1 each). The predominance of cell-free supernatants underscores their therapeutic relevance, as they contain bioactive metabolites (e.g., bacteriocins, organic acids, and enzymes) that contribute to antimicrobial activity, immune modulation, and tissue regeneration. The main findings are available in Table 3.

Among the skin models used, the excisional wound model was the most common (68.2%, *n* = 30), followed by burned skin models (18.2%, *n* = 8). Other models included general wound healing assessments (9.1%, *n* = 4) and diabetic wound models (4.5%, *n* = 2). These models assessed the effects of probiotic, postbiotic, and synbiotic formulations in promoting wound healing, reducing bacterial load, and modulating immune responses. Most studies analyzed used in vivo models (54.9%, *n* = 39), followed by in vitro experiments (43.7%, *n* = 31), which included wound healing tests, antimicrobial assays, and cytotoxicity evaluations. Only one clinical trial was found.

### 2.2. Risk of Bias of Animal Studies with Syrcle Tool

The risk of bias assessment using the SYRCLE tool revealed that all animal studies (*n* = 40) presented unclear sequence generation and allocation concealment, reflecting a lack of transparency in the randomization process. All studies were classified as having a high risk of bias in the domain of blinding of caregivers and researchers, given the absence of blinding in the experiments. Half of the studies also presented a high risk of bias for outcome assessors’ blinding. Conversely, studies had a low risk of bias for incomplete outcome data and selective outcome reporting domains, as authors accounted for these features properly. Most studies lacked transparency in key methodological aspects (as random allocation and baseline group homogeneity), with 32.5% of them (*n* = 13) classified as with high risk of bias. These findings highlight structural weaknesses in preclinical animal studies, particularly in randomization, blinding, and allocation concealment, underscoring the need for better reporting and standardization in this type of research (see Supplementary Materials, Supplementary Table S2).

## 3. Discussion

### 3.1. Formulation Strategies for Enhancing Wound Healing via Skin Microbiome Modulation

The microorganisms can impair healing and increase the risk of systemic infections, making proper management essential to prevent complications and optimize recovery [2,14,16,64,65]. A healthy skin microbiota is essential for effective wound healing. Commensal microorganisms play key roles in immune regulation, support epithelial growth, and prevent the colonization and spread of opportunistic pathogens [66,67]. Probiotic treatments can improve wound healing, especially when microbial diversity is preserved or only slightly disrupted. In these situations, probiotics help bolster beneficial microbial communities, trigger the production of anti-inflammatory cytokines, and facilitate tissue regeneration [39,44]. In instances of severe dysbiosis or active infection, probiotics may act as supportive strategies to control harmful overgrowth, restore microbial balance, and create a conducive environment for wound healing.

Wound infections are often caused by antimicrobial-resistant bacteria, including Gram-positive pathogens such as *Staphylococcus aureus* (MRSA) and Gram-negative bacteria like *Pseudomonas aeruginosa*, *Klebsiella pneumoniae*, and *Acinetobacter baumannii*. Additionally, anaerobic bacteria, including *Clostridium* spp. (Gram-positive) and *Bacteroides* spp. (Gram-negative), contribute to tissue necrosis and further complicate the healing process [68]. Probiotic therapies may help fight antimicrobial resistance by preventing the colonization of multidrug-resistant pathogens, generating antimicrobial substances (such as bacteriocins and lactic acid), and strengthening local immune responses [22,40]. Pre-clinical studies indicate that probiotics could interfere with biofilm formation, lower pathogen levels, and possibly restore antibiotic effectiveness in infected wounds [22,40,61]. While clinical support is still limited, using probiotics as an adjunct in wound care could be a promising approach to enhance infection control and decrease dependence on traditional antibiotics, thus reducing the selective pressures that contribute to the development of resistance.

Fungal infections, such as those caused by *Candida* spp. and *Aspergillus* spp., along with viral pathogens like *Herpes simplex* virus and *Cytomegalovirus*, pose additional challenges, particularly in immunocompromised patients [68,69]. Effective management of these infections requires microbiological surveillance, rational use of antimicrobials, and adjuvant therapies to prevent complications and optimize recovery. Traditionally, microbiological surveillance relies on culture techniques, Gram staining, antibiograms, and colony morphology analysis. However, these methods are unable to detect non-cultivable microorganisms and do not accurately reflect the microbiota changes induced by probiotics. Therefore, the incorporation of molecular tools, such as 16S rRNA gene sequencing or metagenomics, may improve the monitoring of these changes and support more precise therapeutic decisions [70,71].

Advanced topical delivery, like hydrogels, films, and microneedle patches, provide considerable benefits compared to standard ointments and creams for influencing skin microbiota and improving wound healing. Hydrogels maintain a moist, biocompatible setting that safeguards probiotic viability, encourages epithelialization, and enables the controlled, sustained release of bioactive substances. Because of their flexibility and permeability, polymeric films offer mechanical protection while promoting the gradual diffusion of therapeutic agents, supporting stable colonization by beneficial microorganisms. Microneedle patches allow for direct delivery of probiotics or postbiotics into the skin’s deeper layers, bypassing surface barriers and enhancing immune response at the tissue level. In contrast, traditional ointments and creams typically create occlusive, lipid-heavy layers that can hinder probiotic survival, restrict oxygen flow, and diminish controlled release effectiveness. Consequently, innovative delivery systems enhance microbiome-modulating products’ stability and therapeutic efficacy and improve wound bed conditions to speed healing and lower infection risks [19,20,24,27,29,32,33,34,35,36,37,38,39,40,41,42,43,44].

#### 3.1.1. Probiotics Formulations

Probiotics are live microorganisms that provide health benefits to the host when given in sufficient quantities. In addition to their known effects on gut health, specific probiotic strains have shown distinct abilities to influence skin microbiota and enhance wound healing [72,73]. Strains like *L. plantarum*, *L. reuteri*, and *B. bifidum* demonstrate resilience to oxidative stress, adhere strongly to keratinocytes, produce antimicrobial peptides, and regulate cytokine responses [74,75]. Notably, skin strains differ from those for the gastrointestinal tract; they are designed for aerobic, oxidative environments while interacting with epidermal and immune cells. In contrast, gut strains thrive in anaerobic niches and exert their effects primarily through interactions with mucosal immune components such as dendritic cells and IgA-producing lymphocytes [67,75]. Despite these differences, gut and skin probiotics fulfill distinct yet complementary roles [75]. Gastrointestinal probiotics help restore microbiome balance, improve digestion, support nutrient absorption and improve immune systemic immune regulation. Meanwhile, skin probiotics strengthen the epidermal barrier, modulate local immunity, and may alleviate conditions such as acne, atopic dermatitis, and wound-related inflammation [76].

*L. plantarum* stands out for its dual-site activity. It can colonize the gut and, although not a natural skin commensal, adhere transiently to the skin and act topically with antimicrobial and anti-inflammatory effects [25,38,45,48,61,77,78]. *L. reuteri*, although it colonizes the gastrointestinal tract, does not naturally inhabit the skin but can influence skin health through systemic immune and neuroendocrine pathways as well as through topical applications, acting functionally rather than through true colonization [19,32,35,44,79,80,81]. In contrast, *B. bifidum* is an established gut colonizer but does not colonize the skin. Nonetheless, it exerts beneficial effects through its postbiotic metabolites—such as extracellular polysaccharides and cell wall fragments, which have been shown to improve skin hydration, reduce inflammation, and support barrier repair when applied topically or administered orally [21,82]. These distinctions underscore the need for strain-specific selection based on the target site, mechanism of action, and desired therapeutic outcome.

These features are critical for restoring skin barrier integrity, controlling pathogen colonization, and supporting tissue regeneration. The cutaneous administration of probiotics thus represents a promising alternative to conventional antibiotics, particularly for managing both infected and non-infected wounds [19,20,24,27,29,32,33,34,35,36,37,38,39,40,41,42,43,44]. Probiotics facilitate wound healing through different mechanisms, depending on whether an infection is present. Infected wounds primarily function by exhibiting antimicrobial properties, producing compounds such as bacteriocins, organic acids (like lactic and acetic acids), hydrogen peroxide, short-chain fatty acids, and bioactive peptides. These substances impede the growth of harmful microorganisms, compete for nutrients and adhesion sites, and disrupt biofilm formations [61,83,84]. Together, these effects help manage infections and reduce excessive inflammation, aiding the transition to the proliferative healing phase. Conversely, in non-infected wounds, probiotics boost epithelial cell proliferation, encourage angiogenesis, regulate immune responses, and enhance the release of growth factors and anti-inflammatory cytokines. This creates a more supportive environment for tissue regeneration. The two distinct modes of action underscore the adaptability of probiotics as therapeutic agents in varying wound scenarios [9,36,85,86].

In addition, probiotics can modulate the local immune response in wound healing by regulating cytokine production and influencing inflammatory cell recruitment. They have been shown to increase anti-inflammatory cytokines such as IL-10 and TGF-β while reducing pro-inflammatory mediators like TNF-α, IL-6 and IL-1β [39,61]. Additionally, probiotics can promote the polarization of macrophages toward an anti-inflammatory M2 phenotype, supporting inflammation resolution and tissue repair [19]. Table 1 depicts the extracted results and the main findings of the selected studies.

There is a differentiation among probiotic strains and their metabolites in the wound-healing process, as each may act through specific pathways. Although Lactobacillus species commonly produce bacteriocins and lactic acid—compounds that help create a favorable environment for skin regeneration—they also synthesize other metabolites that confer distinct efficacy against various pathogens [74,75]. The type and concentration of these metabolites vary according to the specific probiotic strain, leading to strain-dependent differences in antimicrobial activity [75]. For example, *L. plantarum*, *L. acidophilus*, and *L. reuteri* have demonstrated activity against *S. aureus* and *P. aeruginosa*. In addition, *L. plantarum* has shown effectiveness against *E. coli*, *L. acidophilus* against *Klebsiella* spp., and *L. reuteri* against *Salmonella* [19,20,25,32,44,48,61,77,78,80,87,88,89].

*L. reuteri* and *L. fermentum* modulate local immunity by reducing TNF-α levels and increasing IL-10 expression. Both *L. reuteri* and *Lactococcus lactis* stimulate angiogenesis by VEGF expression. Moreover, *L. lactis* promotes macrophage polarization toward an anti-inflammatory M2 phenotype, thereby contributing to more effective tissue repair in the later stages of wound healing—an especially relevant feature in the management of complex wounds, such as chronic or infected wounds [19,32,35,44,63,80,81]

##### Gels

Gels are an effective delivery system for probiotics in wound healing due to their hydrating properties, bioadhesion, and controlled release, creating a moist environment that promotes epithelialization, collagen deposition, and reduced scar formation [23,30,35]. Their bioadhesive nature ensures prolonged contact with the wound bed, enhancing antimicrobial and immunomodulatory effects. Additionally, gels provide stability and uniform dispersion of probiotic metabolites, such as bacteriocins, organic acids, and exopolysaccharides, which aid in pathogen inhibition and tissue regeneration [41,42,49]. Hydrogels, with their high water content and adaptability to wound shapes, further enhance drug release and integration with the extracellular matrix, making them a widely used option in wound healing [19,49,90].

For instance, Tsai et al. (2021) investigated the effects of a hydrogel formulation (Ulora Gel) at a final concentration of 1 × 10^10^ cells/g of gel containing heat-killed *L. plantarum* GMNL-6 and *L. paracasei* GMNL-653 in a full-thickness tail wound model in BALB/c mice—with wounds treated once daily for five days [36]. The treatment accelerated wound closure, with significant improvements observed by day 3 and day 15 compared to untreated controls. By day 20, nearly complete wound healing was achieved in the probiotic-treated groups, whereas the control group still showed open wounds. Histological analysis revealed that probiotic-treated wounds had increased collagen synthesis, enhanced re-epithelialization, and reduced fibrosis accumulation [36]. Mechanistically, the beneficial effects of heat-killed Lactobacillus strains were attributed to the modulation of TGF-β/Smad signaling, which is critical in fibroblast activity and extracellular matrix remodeling. The probiotics also inhibited excessive myofibroblast differentiation, thereby preventing hypertrophic scarring [91].

Yet, among the various probiotic strains, *L. plantarum* LP-G18-A11 has demonstrated significant potential in reducing pathogenic bacterial load, enhancing skin regeneration, and improving formulation stability in hydroxyethyl cellulose-based gels (Natrosol). A study by Sousa et al. (2023) evaluated the antimicrobial effects of a hydroxyethyl cellulose-based gel containing this strain at 5% and 3% (*w*/*v*) [24]. The probiotic-based gel was tested against *S. aureus* and *P. aeruginosa* using in vitro and ex vivo wound models. The 5% *L. plantarum* LP-G18-A11 gel exhibited the highest antibacterial activity, showing an inhibition zone of 10 mm for *S. aureus* and 17 mm for *P. aeruginosa* [24]. Similarly, in an ex vivo wound infection model using porcine skin, wounds were contaminated with *S. aureus* and *P. aeruginosa* before treatment with the probiotic-based gel. After 24 h, the bacterial load was significantly reduced (31% reduction for *S. aureus* and 52% for *P. aeruginosa*), with results comparable to those obtained with ciprofloxacin treatment (37% and 62%, respectively). However, after 72 h, the reduction was maintained only for *P. aeruginosa*, highlighting the strain’s potential for addressing Gram-negative infections in wounds [24].

The stability of the *L. plantarum* LP-G18-A11 gel was also assessed under different storage conditions. The formulation maintained bacterial viability for up to 90 days at 4 °C; at 25 °C, viability declined after 21 days. The formulation remained stable in preliminary and accelerated stability tests, with no phase separation or significant changes in pH, texture, or odor [24]. Future studies should focus on optimizing storage conditions, evaluating synergistic effects with other bioactive compounds, and conducting in vivo trials to validate clinical efficacy [24].

To assess the potential of an oxidized *Bletilla striata* polysaccharide-chitosan composite (OBSP-CS-LP) hydrogel as a delivery system for probiotics, a hydrogel was formulated by crosslinking 40% oxidized Bletilla striata polysaccharide with 1.5% chitosan (*w*/*v*), incorporating *L. plantarum* at a density of 1.0 × 10^10^ CFU/mL [42]. The hydrogel created a slightly acidic microenvironment, which enhanced fibroblast proliferation, oxygen supply, and bacterial inhibition. It was applied twice daily for 14 days in a full-thickness skin defect model in Kunming mice (18–22 g). The study included a blank group treated with saline (0.9%), an OBSP-CS hydrogel group, and an OBSP-CS-LP hydrogel group treated with L. plantarum. Probiotic-loaded hydrogels significantly accelerated wound closure, with higher collagen deposition, increased angiogenesis (VEGF expression), and reduced inflammation (IL-6, TNF-α levels) compared to the control group (blank group treated with saline (0.9%). Additionally, antimicrobial assays showed that OBSP-CS-LP hydrogel effectively inhibited *S. aureus*, *P. aeruginosa*, and *E. coli*, reducing bacterial colonization by over 98%. The formulation remained biocompatible, with no cytotoxic effects observed in fibroblast cultures, and did not induce systemic toxicity in liver and kidney histological studies [42]. However, comparison with a commercial standard formulation for wound treatment is still necessary for the assessment of its pre-clinical relevance.

Hydrogels containing *L. plantarum* can also be incorporated into dual-layer wound dressing (DLD), demonstrating superior healing efficiency in an infected wound model [38]. The DLD system was designed with an inner hydrogel layer containing *L. plantarum* and an external hydrocolloid layer, which provided mechanical flexibility, bioadhesion, and moisture retention. The hydrogel layer was fabricated using a freeze–thaw method to preserve the viability of heat-sensitive *L. plantarum*. In contrast, the hydrocolloid layer was produced through a hot-melt method, ensuring strong adhesion and elasticity. The optimized formulation consisted of guar gum, polyvinyl alcohol (PVA), and sodium carboxymethyl cellulose, creating a biocompatible, high-swelling, and flexible dressing [38]. Additionally, an in vivo wound recovery study using Pseudomonas aeruginosa-infected Sprague Dawley rats showed that *L. plantarum*-loaded DLD significantly accelerated wound closure compared to a commercial dressing (Duoderm^®^) and an untreated control group. By day 3, the DLD-treated wounds exhibited a 67.8% recovery rate, whereas the commercial dressing and control groups showed only 30.4% and 14.2% recovery, respectively. Histological analysis confirmed complete re-epithelialization and collagen deposition in DLD-treated wounds, while other groups displayed persistent inflammation and incomplete tissue regeneration. DLD provided an antimicrobial effect against *P. aeruginosa*, attributed to producing bacteriocins and organic acids by *L. plantarum*. The hydrocolloid layer enhanced moisture retention, creating a favorable healing environment and facilitating cell migration and tissue repair [38].

In this review, injectable hydrogels were also identified, possessing properties such as self-healing and injectability, which are particularly beneficial for cutaneous wound healing. These characteristics help minimize gel fragmentation and restore its integrity in the affected microenvironment, even after experiencing external mechanical damage, thereby ensuring prolonged and effective support throughout the healing process [90].

The therapeutic potential of a self-healing injectable hydrogel loaded with *L. rhamnosus* (HPF@L.rha) for the treatment of infected full-thickness wounds was investigated by Mei et al. [39]. This formulation was designed using hyaluronate-adipic dihydrazide (HA-ADH), aldehyde-terminated Pluronic F127 (PF127-CHO), and fucoidan (FD), creating a biocompatible, bioadhesive, and extracellular matrix-mimicking scaffold. *L rhamnosus* was incorporated at 1 × 10^7^ CFU/mL, ensuring optimal probiotic viability and prolonged therapeutic effects on the wound site [39]. HPF@L.rha hydrogel was topically applied to infected wounds in Sprague Dawley rats, followed by coverage with sterile gauze to maintain the dressing in place. This intervention demonstrated significantly faster wound closure, enhanced re-epithelialization, and increased collagen deposition compared to untreated control groups and commercial Prontosan-treated wounds. The hydrogel’s moisture-retentive and self-healing properties contributed to its prolonged adhesion to the wound, allowing continuous probiotic release and antimicrobial action. *L. rhamnosus* hydrogel formulation also exhibited strong antibacterial activity against *P. aeruginosa*, reducing bacterial load dose-dependently. Anti-inflammatory properties of the probiotic hydrogel were confirmed by a decrease in pro-inflammatory cytokines (TNF-α, IL-6) and an increase in IL-10 expression [39].

*L. reuteri* has also been investigated for its potential in promoting wound healing and combating infections through hydrogel-based delivery systems. Zhou et al. (2023) developed a metal-phenolic self-assembled hydrogel system that effectively protected probiotics from antibiotic interference while enhancing wound healing and tissue regeneration [19]. The formulation incorporated *L. reuteri* shielded with a metal-phenolic coating (*L. reuteri*@FeTA), where tannic acid and ferric ions (Fe^3+^) formed a supramolecular protective layer around the probiotic. This shielding mechanism adsorbed and inactivated antibiotics, preventing their bactericidal effect on probiotics [19]. The encapsulated *L. reuteri* was then incorporated into an injectable hydrogel matrix composed of carboxylated chitosan (CCS) and oxidized hyaluronic acid (OHA), which provided a moist and bioactive environment for wound healing. This injectable hydrogel was topically applied to full-thickness wounds in BALB/c mice. The wounds were treated with pure L. reuteri, Tegaderm™, and sterilized hydrogels only. The authors considered the gentamicin-treated group as a positive control. When gentamicin was introduced to evaluate probiotic survival and wound recovery, the Gel/L@FeTA-treated wounds exhibited significantly improved healing outcomes, including enhanced angiogenesis (CD31, VEGF expression), reduced inflammation (lower TNF-α, higher IL-10 levels), and increased collagen deposition. Hair follicle regeneration and granulation tissue formation were markedly superior in Gel/L@FeTA-treated wounds [19].

Another study utilized *L. reuteri*-loaded gelatin hydrogel microparticles (10^8^ CFU/mL) prepared via emulsion polymerization of methacrylate-modified gelatin under light irradiation. The study evaluated a negative control (wound covered with gauze only), the HA group (hydrogel with microspheres without *L. reuteri*), and the LRHA group (hydrogel containing *L. reuteri*). This formulation, topically applied to BALB/c mice, accelerated wound healing in *S. aureus*-infected wounds, promoting faster re-epithelialization, increased collagen deposition, and reduced inflammation compared to control groups. However, no standard treatment was used to compare the efficacy of this probiotic-loaded hydrogel formulation, limiting the ability to contextualize its therapeutic performance against clinically established wound care options. Additionally, the hydrogel was developed as an injectable formulation for direct application to infected wounds, enabling the controlled release of beneficial metabolites, including lactic acid and antibacterial agents produced by *L. reuteri*, to modulate the wound microbiome [35].

Therapeutic potential of a multifunctional hydrogel loaded with *Lactococcus lactis* for treating diabetic full-thickness wounds was also identified. The hydrogel formulation, designed using a heparin-poloxamer (HP) thermoresponsive hydrogel, provided a bioactive scaffold to support probiotic viability and function. *L. lactis* was incorporated into the hydrogel, allowing for controlled bacterial retention and sustained vascular endothelial growth factor (VEGF) production, which played a key role in stimulating angiogenesis and enhancing wound repair [41]. When topically applied to full-thickness wounds in diabetic mice, followed by sterile dressing coverage, the *L. lactis*-loaded hydrogel significantly improved wound closure, enhanced neovascularization, and reduced inflammation, compared to untreated control groups and standard wound dressings. The hydrogel’s moisture-retentive properties contributed to prolonged probiotic activity, ensuring continuous VEGF release and immune modulation. Additionally, *L. lactis* influenced macrophage polarization, shifting them from pro-inflammatory to an anti-inflammatory phenotype, promoting a balanced immune response and tissue regeneration [41].

Multi-compartmented microgels loaded with *L. fermentum* and deferoxamine (DFO—an iron chelator used in iron overload-related diseases due to its antioxidant, anti-inflammatory, and angiogenic properties) have demonstrated positive effects for the treatment of multidrug-resistant *P. aeruginosa* (MPA)-infected wounds [40]. The microgel formulation developed by electrospray and microfluidics technology has a dual-compartment hydrogel system where *L. fermentum* and DFO were separately encapsulated within calcium alginate-based microgels. This structure can protect probiotics from antibiotics, ensuring their viability, while providing a controlled release of DFO to enhance angiogenesis and wound healing [40]. After topically applying probiotic-loaded microgel to the full-thickness wounds infected with MPA, followed by coverage with sterile gauze, a significant reduction in bacterial infection (87.44% inhibition of MPA) and improved angiogenesis (29.32% increase in vascularization) were observed. It accelerated wound healing, compared to untreated and antibiotic-treated groups. The microgel’s moisture-retentive and bioadhesive properties contributed to prolonged probiotic viability and antimicrobial activity at the wound site [40].

Finally, emulgels—topical drug delivery systems that combine the properties of emulsions and gels—can offer greater spreadability, prolonged drug release, and improved bioavailability for wound management [33]. The in vivo study by Rasheed et al. (2020) [33] evaluated the therapeutic potential of an emulgel containing *L. acidophilus* HT1 in an infected wound healing model using white rabbits. The rabbits were infected with Pseudomonas aeruginosa, *S. aureus*, or *Klebsiella* spp. After treatment applied twice daily for seven days, the probiotic-treated groups showed faster wound closure, increased collagen deposition, and significant bacterial inhibition. The sustained release of bacteriocins (320 AU/mL) from *L. acidophilus* HT1 ensured prolonged antimicrobial activity.

##### Ointments

Ointments are effective for wound healing due to their moisturizing properties and protective barrier against infections or external factors. Its oily base allows the gradual release of bioactive compounds, such as antimicrobials and anti-inflammatory agents, promoting cellular regeneration and inflammation control. Additionally, the ointment is easy to apply, comfortable, and provides a favorable environment for healing, making it especially useful for superficial and moderately deep wounds [32,92,93].

Probiotics in topical formulations have been widely studied as an innovative approach to wound treatment due to their antimicrobial, anti-inflammatory, and tissue-regenerating properties. Probiotic ointments, particularly those containing Lactobacillus species, have been shown to accelerate wound healing and reduce pathogen colonization in various experimental models. Topical application of probiotics promotes the elimination of pathogenic microorganisms and stimulates skin regeneration [27,32,93].

The beneficial effects of probiotic ointments are attributed to several mechanisms that contribute to wound healing and infection control. These formulations promote the production of antimicrobial substances, such as bacteriocins and organic acids, which effectively inhibit the growth of opportunistic pathogens. Additionally, probiotic ointments play a role in modulating the inflammatory response, as they help reduce pro-inflammatory cytokines while simultaneously increasing growth factors essential for tissue repair and regeneration. Another key benefit is the stimulation of collagen deposition, which enhances extracellular matrix organization and accelerates re-epithelialization, ultimately leading to faster and more effective wound healing [27,32,37,53].

Horikawa et al. (1986) [27] investigated the effects of an ointment containing *L. casei*, composed of Solbase and *L. casei*, on thermal wound healing and bacterial clearance in mice. The study revealed that the ointment accelerated healing in uninfected and P. aeruginosa-infected burns and wounds infected with a mixture of *S. aureus*, *E. coli*, and *P. aeruginosa*. Collagen fiber formation in the subcutaneous tissue treated with *L. casei* was more pronounced than in the group treated only with the ointment base (Solbase). Compared to conventional treatments such as Eksalb and Azunol ointments, mafenide acetate, and silver sulfadiazine (Geben) creams, the *L. casei* ointment demonstrated superior or equivalent effectiveness in bacterial elimination and wound healing enhancement. These effects were further potentiated by adding gentamicin or ofloxacin [27].

Although the study did not directly compare different types of bases (e.g., oily vs. water-soluble), the choice of vehicle can influence the release and efficacy of probiotics. Oily bases such as Solbase may offer slower release of active compounds, while water-soluble bases may facilitate faster release. Therefore, vehicle selection should take into account the desired release characteristics and the stability of probiotics in the formulation [94,95].

Recent studies also reinforce the benefits of probiotic ointments in reducing inflammation and accelerating wound closure. Khodaii et al. (2019) demonstrated that a *L. reuteri*-based ointment significantly reduced myeloperoxidase (MPO) enzyme activity, an inflammatory marker, while increasing collagen deposition and wound re-epithelialization. The wound contraction rate was significantly higher in probiotic-treated groups compared to controls [32].

Furthermore, Kazemi et al. (2022) reported that metabolites from *L. plantarum* and *L. casei* effectively improved cell viability and wound healing, particularly when combined with mesenchymal stem cells (MSCs). The study showed that the topical application of these metabolites significantly accelerated wound closure in a murine model infected with *P. aeruginosa* [37]. The efficacy of probiotic ointments has also been compared with conventional topical antibiotics. Moghadam et al. (2020) used a *L. plantarum*-based ointment to treat *P. aeruginosa*-infected wounds in rats. The results showed that probiotic-treated groups had smaller wound sizes and better tissue repair than those treated with imipenem. Inflammation was also lower in probiotic-treated groups compared to both the control and antibiotic-treated groups [53].

##### Powder Microparticles

Dry powder formulations for wounds consist of microparticles or nanoparticles designed to adhere to injured tissue, creating an environment conducive to healing. These systems offer advantages such as ease of application, exudate absorption, bacterial protection, and controlled release of bioactive compounds. In contrast to creams or gels, powder formulations do not require liquid carriers, minimizing microbial contamination and improving the stability of active ingredients.

Ben David et al. (2021) [34] developed a polyvinyl alcohol (PVA) microparticle formulation containing *B. subtilis*, a microorganism capable of secreting antimicrobial compounds and promoting tissue regeneration. These microparticles, produced by spray-drying, had an average diameter of 4.5 μm and were designed to adhere to injured tissue without causing irritation or adverse effects. This dry powder formulation was applied directly to open wounds in C57BL6 mice, where it remained until fully dissolved, creating a moist environment that supported healing and enabled the sustained release of antimicrobial molecules from *B. subtilis*. Treated wounds showed accelerated closure compared to the control group (without treatment), leading to complete tissue regeneration within 12 days.

In in vitro experiments, the dry powder formulation exhibited high antimicrobial efficacy against *S. aureus* and MRSA, attributed to the controlled release of surfactin and fengycin, which act as antimicrobial agents. These findings suggest that encapsulating probiotic bacteria in polymeric systems may be a promising strategy for treating complex and antibiotic-resistant wounds.

Although the study by Ben David et al. 2021 [34] provides promising evidence for the use of *B. subtilis* in spray-dried PVA microparticles for wound treatment, it remains the only report currently addressing this specific formulation. Further studies are needed to explore additional probiotic strains and delivery systems—such as alginate-, cellulose-, or nanoparticle-based matrices—to strengthen the existing evidence base. The lack of comparison with standard treatments, such as antibiotic ointments, limits conclusions regarding its preclinical superiority. While no adverse effects or skin irritation were observed in mice over a 15-day period, detailed toxicological and immunological safety assessments were not conducted. Moreover, while spray-drying is associated with extended shelf life, the long-term viability of the *B. subtilis* formulation was not assessed. These gaps highlight the need for additional research to validate its efficacy, stability, and safety in both preclinical and clinical settings.

##### Microbeads

Microbeads are polymeric carriers designed for encapsulation, protection, and controlled release of bioactive agents. These engineered delivery systems can be tailored for precise degradation kinetics, diffusion properties, and environmental responsiveness, ensuring optimized therapeutic efficacy. In the study by Jones et al. (2012) [29], alginate-based cross-linked microbeads were produced via ionic gelation with CaCl_2_, followed by lyophilization, encapsulating *L. fermentum* NCIMB 7230, a probiotic strain that generates gaseous nitric oxide (gNO) through metabolic conversion of glucose and nitrite salts. These lyophilized microbeads, combined with a solution containing 10% (*w*/*v*) glucose, 0.85% (*w*/*v*) NaCl, and 30 mM sodium nitrite, were incorporated into gas-permeable adhesive patches (Tegaderm™), enabling on-demand NO release [29]. Using a New Zealand white rabbit model of ischemic and infected full-thickness wounds, the authors demonstrated that these patches enhanced wound closure, increased angiogenesis, and improved collagen deposition. This probiotic-driven NO release system provided antimicrobial activity against *S. aureus*, highlighting the dual role of microbeads as bioreactors for NO release and structural carriers for controlled probiotic delivery [29].

Although the study by Jones et al. (2012) [29] presents promising results for a nitric oxide (NO)-releasing probiotic patch incorporating *L. fermentum*, the patch accelerated healing in ischemic and infected wounds through controlled NO release, however, was only compared to a vehicle control, with no evaluation against standard treatments such as antibiotic ointments. This study did not report on the long-term viability or stability of *L. fermentum* following lyophilization or storage.

Since *L. fermentum* was primarily used as a biocatalyst for NO production, it remains unclear whether the observed therapeutic effects are due to the probiotic itself, the released NO, or a combination of both. These limitations underscore the need for further studies exploring different probiotic strains and delivery platforms—such as polymeric carriers—to strengthen the evidence base. Moreover, future investigations should include direct comparisons with standard treatments and comprehensive safety assessments to support clinical translation.

##### Films

Polymeric films are typically thin, flexible, and biocompatible materials made from either natural or synthetic polymers. Polymeric films have emerged as versatile wound-dressing materials, offering controlled bioactive release, antimicrobial activity, and mechanical support. In this review, a study with calcium alginate-based films incorporating *L. plantarum* demonstrated effective antimicrobial properties against *S. aureus* and *P. aeruginosa*, with high probiotic viability sustained for up to six months at 4 °C [28]. However, these films loaded with *L. plantarum* were not directly compared with conventional wound treatments, such as gauze or topical antibiotics, which limits the contextualization of their future clinical benefits.

Similarly, coaxial electrospun nanofiber films composed of polycaprolactone (PCL) and silk fibroin supported the formation of *L. paracasei* biofilms, enabling continuous probiotic colonization and prolonged antibacterial action [43].

These films’ physicochemical and mechanical properties play a crucial role in their therapeutic performance. Alginate films exhibit high moisture retention and rapid biodegradability, making them suitable for short-term applications that require fast bioactive release. In contrast, PCL-based nanofiber films offer prolonged degradation, allowing long-term wound protection and structural integrity. These films’ flexibility and tensile strength further enhance wound adaptability, ensuring optimal interaction with the skin and efficient bioactive delivery.

Studies in animal wound models demonstrated the therapeutic benefits of probiotic-loaded films, highlighting their effectiveness in infection control and wound healing. In a full-thickness wound model using Wistar rats, *L. plantarum*-loaded alginate films significantly reduced bacterial load, accelerated wound closure, and improved epithelial regeneration, as confirmed by histological analysis showing increased collagen deposition and reduced inflammation [28]. Similarly, in a murine (BALB/c mice)-infected wound model, electrospun PCL-silk fibroin films promoted angiogenesis (CD31 expression), tissue remodeling, and immune modulation by decreasing pro-inflammatory cytokines (IL-6, TNF-α). The PCL-based films outperformed the commercial nano-silver burn and scald dressing (Anson^®^) in terms of wound-healing efficacy, antimicrobial activity, and tissue regeneration [43].

Edible films loaded with probiotics have also been investigated as consumer-acceptable alternative to conventional systems. Various strains, such as *L. plantarum*, *Bifidobacterium animalis* subsp. *lactis BB-12*, and *L. acidophilus*, have been incorporated into alginate, starch, and kefiran matrices (polysaccharide produced by lactic acid probiotics). These formulations showed greater antimicrobial activity, improved probiotic viability, and, in some cases, more effective inhibition of pathogens such as *Listeria monocytogenes* and *P. aeruginosa* compared to films without probiotics [96]. These findings reinforce the functional role of probiotics in wound treatment films and highlight the importance of proper selection of both the strain and the polymeric matrix.

##### Scaffolds

Probiotic-functionalized nanocomposite scaffolds are emerging as an innovative strategy for infection control and tissue regeneration in skin wounds, mainly burns. Scaffolds are three-dimensional biomaterial structures that aid tissue regeneration by imitating the extracellular matrix. These systems offer a physical framework that supports cell adhesion, proliferation, and migration, allowing for the controlled release of therapeutic agents such as probiotics, antimicrobials, or growth factors. Scaffolds improve tissue repair during wound healing by facilitating re-epithelialization, angiogenesis, and collagen deposition, especially in challenging wounds like burns [97].

Khan et al. (2019) [31] developed scaffolds through the electrospinning technique using poly (vinyl alcohol) (PVA), poly(vinylpyrrolidone) (PVP), and glycerol, incorporating the probiotic bacterium *E. mundtii* QAUEM2808. This approach enabled efficient probiotic encapsulation into nanostructured fibers (~318 nm), ensuring their viability for up to four weeks and promoting controlled degradation in simulated wound fluid, facilitating the sustained release of the microorganism. These scaffolds exhibited antimicrobial activity against pathogens such as *S. aureus* and *P. aeruginosa*, as well as significantly accelerating burn wound healing in BALB/c mice by stimulating epithelialization, collagen deposition, and hair follicle formation. These findings suggest that functionalizing scaffolds with probiotics may represent a novel approach to wound treatment, combining the benefits of tissue bioengineering with microbiome modulation [31].

Another innovative approach combines collagen scaffolds incorporated into a hydrogel for the topical application of probiotics in new dressing methods, aiming to protect burn wounds from infections and accelerate the healing process [30]. The topical application of a collagen hydrogel/scaffold containing *S. cerevisiae* (10^7^ CFU/mL) on sterile cotton gauze dressing significantly increased collagen content, promoting the healing of cutaneous burn wounds compared to untreated wounds in Sprague Dawley rats (200–250 g). After 22 days of treatment, the degradation of the collagen hydrogel scaffold was observed, with no evidence of an immune reaction and without signs of infection in the treated animals. The presence of the collagen hydrogel in the formulation was essential, as it provided the necessary moisture to activate *S. cerevisiae*, optimizing its therapeutic effect [30]. The hydrogel loaded with an *S. cerevisiae* collagen scaffold demonstrated increased collagen content, improved biomechanical properties, accelerated epithelialization, and microbial inhibition.

##### Other Formulations

Studies investigating less conventional topical delivery systems were also identified. A probiotic-loaded microneedle MN patch, produced using polyvinyl alcohol (PVA) and sucrose, was designed to encapsulate *L. reuteri*, a probiotic strain with antimicrobial properties. The MN patches were fabricated through molding and vacuum-assisted drying, ensuring mechanical strength for skin penetration while allowing rapid dissolution upon application. *L. reuteri* generates reuterin, providing broad-spectrum antimicrobial activity against *S. aureus*, *E. coli*, and *P. aeruginosa* [44]. The 5% Glycerol micro needle patch exhibited antibacterial and anti-inflammatory effects by inhibiting *S. aureus* growth and reducing toxin secretion. It downregulated CD86 expression in RAW 264.7 cells and significantly decreased IL-6 and TNF-α levels, highlighting its therapeutic potential for infected wound treatment. This device was engineered to ensure structural integrity for skin penetration, followed by rapid dissolution upon application, allowing sustained therapeutic action. The system demonstrated high probiotic viability (>80%) for up to 60 days under refrigeration. Additionally, a mouse model of infected full-thickness wounds showed accelerated wound closure, increased collagen deposition, and reduced inflammation compared to untreated wounds by the use of this device [44].

##### Formulation Stability

The stability of probiotic microorganisms plays a vital role in their therapeutic effectiveness, particularly in topical applications where environmental factors could compromise their viability. Several intrinsic and extrinsic elements—such as temperature, humidity, oxygen exposure, pH, and mechanical stress—can influence probiotics’ survival during formulation, storage, and application [98,99]. Consequently, the design of formulations is essential for maintaining probiotic stability. Gels and hydrogels create a moist, buffered, and semi-occlusive environment that helps retain bacterial viability while allowing controlled release at the wound site [35,100,101]. Emulgels and microencapsulated systems (like microparticles and microbeads) provide additional safety by physically enclosing the microorganisms within polymeric matrices, protecting them from oxygen and shear forces during processing and application [34]. Freeze-dried or lyophilized probiotics in films [22,25,26] or microneedles can remain viable for long durations when kept at low temperatures, ensuring shelf stability [44,62,102]. Selecting biocompatible polymers, adding cryo/lyoprotectants, and optimizing hydration levels are crucial formulation factors that improve probiotic survival. Therefore, the therapeutic effectiveness of topical probiotic systems relies not just on the choice of strain and dosage but also on the formulation’s capability to maintain viability and ensure targeted delivery to the wound bed.

#### 3.1.2. Synbiotic Formulations (Live Microorganisms with Prebiotics)

Recent advancements in wound healing have shown the potential of synbiotic formulations, combining probiotics and prebiotics, to address infection control and tissue regeneration. These innovative approaches leverage diverse delivery systems to enhance therapeutic efficacy, stability, and compatibility of synbiotic components, making them highly effective in complex wound environments.

As demonstrated in Table 2, different topical formulations have been developed to deliver synbiotics to the skin. One promising development is using microecological hydrogels, incorporating *L. plantarum* and FOS. These hydrogels provide a supportive environment for beneficial microbial growth, eradicate pathogens, and reduce inflammation via lactic acid production, showing superior performance compared to the commercial dressing Silverex^®^ Heal. By modulating skin microbiota and supporting immune responses, especially in diabetic wounds, this formulation demonstrates excellent integration of microbiota-modulating properties with immune benefits [49].

Similarly, lyophilized polymeric particles have shown great promise in stabilizing probiotics and prebiotics for direct wound application. Encapsulation of *L. plantarum* within cationic and anionic polymeric matrices resulted in particles with exceptional swelling capacity (approximately 2000%) and sustained probiotic viability exceeding 82%. The freeze-drying process ensured long-term stability under refrigerated conditions, while antimicrobial evaluations revealed efficacy comparable to silver sulfadiazine. This formulation not only supports infection clearance but also enhances wound closure rates [22].

Another innovative approach involves the integration of solid lipid nanoparticles (SLNs) into a film used as a dressing. By co-incorporating curcumin-loaded SLNs and *L. plantarum*, these dressings deliver enhanced antimicrobial activity, with a 560% improvement against *Staphylococcus aureus* biofilms. The sponge dressings are engineered for controlled curcumin release, optimal water vapor transmission, and high tensile strength. Their ability to reduce pro-inflammatory markers and accelerate wound closure highlights their suitability for managing chronic and infected wounds, demonstrating superior performance compared to the commercial dressing Silverex^®^ Heal. The study also analyzes the individual contribution of curcumin and *L. plantarum* in the treatment of chronic and infected wounds, demonstrating a synergistic effect that enhances the antimicrobial and anti-inflammatory activity of the formulation [48].

Freeze-dried dressings loaded with Vitamin E and *L. plantarum* provide another sustainable and effective wound care example. These dressings, utilizing alginate-based emulsions on Spanish Broom fibers, demonstrated sustained release over 24 h, high antioxidant activity, and robust antimicrobial properties against *S. aureus* and *P. aeruginosa*. However, this study did not use a commercial dressing as a comparator, which limits the extrapolation of the clinical benefits to practical application [45].

In addition to these formulations, a study showed that topical gel incorporating the ethyl acetate extract from marine synbiotic *B. amyloliquefaciens* within a macroalgal polysaccharide improved wound closure rates and fibroblast viability, while remaining stable (retention of physicochemical properties) for up to 18 months. The long shelf-life and biocompatibility of this formulation make it particularly appealing for clinical applications in managing multidrug-resistant infections [46]. In another study, an innovative delivery system by instant protection spray (IPS), which combines probiotic extracts with flavones to form a rapid gelation barrier within 30 s, forming a hydrogel film on the skin surface, effectively sterilized multidrug-resistant *S. aureus* both in vitro and in vivo, while reducing inflammation and accelerating healing in burn wounds. However, the individual effect of the flavones or the probiotic bacterium was not demonstrated, making it difficult to specifically attribute the efficacy to a single component [47].

Finally, novel synbiotic hydrogels (HAEPS@L.sei) integrating probiotic (*L. paracasei* TYM202) and prebiotic (extracellular polysaccharide EPS-M76 from *B. velezensis*) components may offer a microbiome-friendly strategy for wound healing [50]. While *L. paracasei* actively produces lactic and acetic acids (suppressing pathogenic bacteria such as *S. aureus* and *E. coli* while maintaining the natural Firmicutes-Proteobacteria balance of the skin microbiota), EPS-M76 serves as a prebiotic substrate, promoting probiotic viability and bioactive compound release. Instead of traditional antimicrobial dressings that indiscriminately eliminate both beneficial and harmful bacteria, this synbiotic formulation preserves microbial diversity, preventing opportunistic pathogen overgrowth and supporting an optimal healing environment [50]. The hydrogel matrix, composed of hyaluronic acid methacrylate and dopamine-modified EPS-M76, ensures biodegradability, injectability, and moisture retention, which is critical for effective wound healing. A model using Sprague Dawley rats with full-thickness wounds confirmed accelerated wound closure (96.47% by day 14), enhanced collagen deposition, increased angiogenesis (VEGF-α expression), and reduced inflammatory cytokines (TNF-α, IL-6) of this formulation compared to saline-treated and non-synbiotic control groups [50].

However, despite these advancements, several gaps remain. Long-term microbial viability under various storage conditions requires further exploration, as does the standardization of in vivo testing to ensure consistent comparisons across formulations. Additionally, the economic feasibility of scaling up these advanced delivery systems for widespread clinical use has not been extensively evaluated [47].

#### 3.1.3. Postbiotic Formulations (Nonviable Components or Metabolites)

Postbiotics, which are defined as nonviable microbial cells or their metabolites, have garnered attention for their significant potential in wound care. Unlike live probiotics, postbiotics offer enhanced stability, which reduces the regulatory challenges associated with using live microorganisms, thereby facilitating their integration into clinical practices and commercial products [103,104]. The unique properties of postbiotics, including their ability to modulate immune responses and promote healing, make them particularly well-suited for wound care applications [26,105], as demonstrated in Table 3.

We found studies demonstrating different wound healing mechanisms driven by postbiotics. For instance, they may accelerate the migration of immune cells (e.g., neutrophils, macrophages) to wound sites by activating pattern recognition receptors (PRRs) such as Toll-like receptors (TLRs), leading to downstream signaling pathways that increase chemokine and cytokine expression (e.g., IL-8, MCP-1), which in turn recruit immune cells to the site of injury [26]. In addition, they aid the fibroplasia process (quicker deposition of collagen and elastin) [106] and produce metabolites such as short-chain fatty acids and extracellular polysaccharides [56,107]. Their antimicrobial properties also support their use, which helps prevent infections at wound sites. This is especially important for treating chronic wounds, as infections can significantly hinder the healing process [26,56,104,105,106]

Creams and gels have been extensively studied as delivery systems for postbiotics due to their ease of application and ability to form a protective barrier over the wound. The first provides an occlusive layer that retains moisture and helps create a favorable environment for wound healing, while gels offer a high water content that supports hydration and cooling of the wound site, which can be particularly beneficial in acute and inflammatory wounds. Van Staden et al. (2016) demonstrated that lysates of *L. acidophilus* in cream formulations significantly reduced bacterial colonization and inflammation [84]. Gels developed by Zouari et al. (2016) using *B. subtilis* lysates also showed strong antibacterial effects against multidrug-resistant bacteria and accelerated wound healing while promoting re-epithelialization [51].

Similarly, Sinha et al. (2019) [55] investigated the efficacy of a postbiotic gel formulation containing *Lactobacillus* (VITSAMJ1) isolated from goat milk. The gel was prepared using 25 mL of bacterial supernatant mixed with glycerin as an emulsifying agent and glycerol, ensuring a stable and bioactive formulation. The treatment protocol involved the cutaneous application of the postbiotic gel twice daily on full-thickness excisional wounds in female Wistar rats (~120 g). The postbiotic gel formulation significantly enhanced wound contraction from day 3 onwards, leading to faster tissue regeneration and reduced wound area. The negative control group received a mixture of glycerin and glycerol—the same vehicle used in the probiotic gel formulation. The inclusion of this group allowed for isolating the effects of the vehicle alone, which showed no significant improvement in wound healing compared to the untreated control. This confirms that the observed therapeutic benefits, including accelerated wound closure and improved histological regeneration, are attributable to the probiotic and not to the vehicle. Histopathological analysis confirmed increased immune cell infiltration, particularly neutrophils and macrophages. By day 11, wounds treated with the postbiotic gel exhibited nearly complete re-epithelialization, whereas control groups showed delayed tissue repair. A transient increase in leukocyte count was observed in the group treated with the postbiotic gel, peaking at approximately 15,000 cells/μL on day 3. This elevation was not detected in the control groups (vehicle and untreated), and leukocyte levels in the postbiotic-treated group returned to baseline values (~6000 cells/μL) by day 4. This temporary rise in leukocytes is indicative of a regulated and beneficial inflammatory response, which likely contributed to accelerated wound closure and improved histological architecture [55]. These findings support the interpretation that the leukocyte elevation reflects immunomodulatory activity rather than an excessive or pathological inflammatory reaction [55,108]. Additionally, the probiotic supernatant exhibited antibacterial activity in vitro against *S. aureus* (MTCC 3160), a key pathogen in wound infections [55].

A recent study evaluated the efficacy of a gel formulation containing the cell-free supernatant of *L. plantarum* (MTCC Lp2621) in excision wound-healing models in BALB/c mice. The gel was formulated using 2% carboxymethyl cellulose (CMC) as the gelling agent, ensuring a stable and bioactive delivery system. The postbiotic was used at a concentration of 1 × 10^9^ CFU/mL for supernatant extraction. The topical application of Lp2621 gel was performed twice daily for 21 days, and its efficacy was compared to vehicle control (CMC alone) and betadine^®^-treated groups. The Lp2621-treated group demonstrated enhanced wound contraction, with significantly higher fibroblast proliferation, vascularization, and re-epithelialization compared to controls. By day 14, the histopathological analysis revealed improved collagen deposition and reduced inflammatory cell infiltration. Additionally, wounds infected with *S. aureus* showed a higher healing rate in the Lp2621 group compared to betadine treatment [20]. A notable mechanism observed in Lp2621-treated wounds was the upregulation of IL-6 in the early inflammatory phase, which facilitated immune activation and pathogen clearance. In the later phase, increased IL-10 expression contributed to anti-inflammatory effects, allowing efficient tissue remodeling and reduced scar formation [20].

Another gel-based formulation containing *L. plantarum* MTCC 2621 has demonstrated antimicrobial, biofilm inhibition, and immunomodulatory properties. This formulation, prepared with 2% carboxymethyl cellulose gel in an MRSA-infected excisional wound model in BALB/c mice, has shown promise in wound healing and infection control. The gel applied twice daily demonstrated significant wound contraction by day 7 and accelerated healing by day 14, with increased fibroblast proliferation, enhanced vascularization, and improved epidermal regeneration. The probiotic supernatant exhibited anti-MRSA activity, with an 11.66 mm inhibition zone, and showed 77% biofilm inhibition at high concentrations. Additionally, Lp2621 treatment modulated inflammatory responses, reducing IL-6 (pro-inflammatory) and increasing IL-10 (anti-inflammatory), contributing to controlled inflammation and tissue remodeling [61].

Film and hydrogel formulations have also been explored for postbiotic delivery, focusing on sustained release and wound protection. Jamaran et al. (2021) [26] developed a postbiotic film combining *L. reuteri* lysates with chitosan, demonstrating robust antimicrobial activity and promoting wound closure in an infected rat model. Shokatayeva et al. (2021) [57] introduced a gel film enriched with *B. subtilis* postbiotics, which reduced healing time by approximately 20% compared to controls. Similarly, Nazari et al. (2024) developed a fiber–hydrogel dressing enriched with postbiotic compounds, emphasizing the role of biomaterial compatibility in enhancing fibroblast adhesion and wound healing [58].

Another novel strategy for wound healing involves the development of bacteriomimetic hydrogels containing membrane vesicles (MVs) derived from postbiotic *L. plantarum* and *L. casei*. This cell-free approach aims to overcome the limitations of live probiotic applications, particularly in immunocompromised patients, while preserving the beneficial effects of probiotics in tissue regeneration [60]. The bacteriomimetic hydrogel was formulated by embedding synthetic microparticles coated with MVs into a hydroxyethyl cellulose (HEC) hydrogel matrix, presenting biocompatibility, bioadhesion, and sustained release properties, ensuring prolonged interaction with the wound site, facilitating anti-inflammatory and regenerative effects. In a full-thickness wound model in mice, the MV-loaded hydrogel significantly improved wound-healing outcomes, reducing inflammation, scar formation, and wound width compared to controls. The hydrogel also modulated the immune response, increasing the IL-10/TNF-α ratio, which is associated with enhanced tissue repair and reduced chronic inflammation. Histological analysis confirmed higher collagen deposition and re-epithelialization in treated wounds [60].

The high-water retention capacity and bioadhesiveness of the chitosan nanogels provide a moist healing environment, further accelerating tissue repair. Incorporating postbiotic lysates into chitosan nanogels is an alternative approach to enhance tissue regeneration and infection control in wound management. Ashoori et al. (2020) developed and evaluated chitosan-based nanogels loaded with probiotic supernatants from *L. reuteri*, *L. fermentum*, and *B. subtilis* sp. natto [18]. These postbiotic-loaded nanogels were formulated using 1 mg of lyophilized probiotic supernatant per 10 g of chitosan nanogel (final concentration: 1% *w*/*w*) and tested in an excisional wound model in Sprague Dawley rats (200–300 g) [18]. All formulations enhanced wound healing, but the one containing *B. subtilis* sp. natto exhibited the best healing quality, as confirmed by histopathological analysis. The postbiotic nanogels improved epithelialization, reduced inflammation, and increased collagen deposition, leading to faster wound closure compared to the untreated group and the chitosan nanogel control group [18].

As another formulation alternative, lotions provide a fluid medium for delivering postbiotics, making them ideal for covering large wound areas with minimal irritation. Ekrami et al. (2024) [59] formulated a lotion containing lysates of *L. rhamnosus* and *B. coagulans* that was effective for reducing wound size and improving skin barrier function, with high patient compliance and no reported adverse effects.

A core–shell microneedle (CSMN) patch, composed of hyaluronic acid and methacrylate gelatin, was designed to sequentially release tannic acid-magnesium (TA-Mg) complexes and extracellular vesicles from *L. druckerii* (LDEVs). The outer TA-Mg shell provided immediate antibacterial action against *S. aureus* and *E. coli*, reduced reactive oxygen species (ROS), and modulated inflammation at the wound site. The inner core, loaded with LDEVs, facilitated keratinocyte and fibroblast proliferation, enhanced angiogenesis (CD31 expression), and promoted collagen deposition, contributing to faster tissue regeneration [62]. The physicochemical and mechanical properties of the CSMN patch were optimized to ensure sufficient penetration force for transdermal delivery, biodegradability, and controlled bioactive release. The system’s biphasic release profile enables early infection control followed by sustained regenerative effects. In the BALB/c mice full-thickness infected wound model, the therapeutic efficacy of the CSMN@TA-Mg/LDEV microneedle patch was confirmed. This advanced system exhibited accelerated wound closure, with complete epithelial regeneration observed by day 21. Histological analysis revealed increased collagen deposition, enhanced epidermal thickness, reduced inflammation (IL-6, TNF-α), and improved skin elasticity [62].

Finally, in an excisional wound model in nude mice, the postbiotics nisin, clausin, and amyloliquecidin, derived from *B. amyloliquefaciens* and applied directly to the wounds (without formulation), demonstrated preliminary antibacterial activity against *Staphylococcus aureus*, including resistant strains, with efficacy equivalent to the commercial mupirocin ointment (GlaxoSmithKline, Research Triangle Park, NC, USA), which should be further evaluated [84].

Although postbiotic formulations delivered by different cutaneous systems may benefit wound management, further studies are needed to optimize these formulations and establish standardized protocols for clinical use. To facilitate clinical translation, several formulation elements need further enhancement. Key areas for improvement include ensuring the long-term viability of probiotics under ambient storage conditions, boosting the physical and chemical stability of the formulations, and enhancing bioadhesive and occlusive properties to promote better skin retention. Moreover, it is vital to optimize release kinetics to sustain therapeutic concentrations at the wound site. Attention also needs to be given to the scalability of production processes and their compatibility with sterilization methods to ease the path toward regulatory approval. Additionally, establishing standardized testing protocols—such as in vitro–in vivo correlation models, biocompatibility tests, and shelf-life evaluations—is crucial for ensuring reproducibility and adherence to regulatory standards.

### 3.2. Clinical Evidence of Postbiotic and Probiotics Formulations for Wound-Healing Therapy

This review identified only one randomized, double-blind, vehicle-controlled clinical trial. Hausmann et al. (2019) investigated the effects of a topically applied *L. lactis* emulsion on skin hydration, barrier integrity, and microbiota modulation in 21 healthy female volunteers with Fitzpatrick skin types II and IV [63]. These volunteers received *L. lactis* emulsion twice daily for 30 days. Results showed an increase in filaggrin and human β-defensin-2 expression, supporting epidermal differentiation and antimicrobial defense, while reducing transepidermal water loss (TEWL) by 18%, confirming its barrier-enhancing properties. Additionally, the formulation optimized skin hydration and stabilized pH, creating a healthier skin microenvironment, with no signs of irritation or adverse effects, demonstrating good biocompatibility. These findings suggest that *Lactococcus*-based formulations could serve as a novel, microbiome-friendly approach to improving skin barrier function, with potential applications in dermatological products [63].

Other clinical studies have demonstrated the therapeutic potential of probiotics in wound healing, even without an optimized dermatological formulation. The clinical trials by Peral et al. (2009, 2010) investigated the direct application of *L. plantarum* to burn wounds and chronic venous ulcers [77,87].

In the first study (Peral et al. 2009) [77], 23 patients (16 males, 7 females, aged 20–60 years) with second- and third-degree burns covering up to 20% of total body surface area (TBSA) were included. The experimental group received *L. plantarum* suspension twice daily, while the control group was treated with silver sulfadiazine (SD-Ag), the standard antimicrobial for burns. The results showed comparable infection control to SD-Ag but with superior granulation tissue formation and faster epithelialization in the probiotic-treated group. No adverse effects were reported, suggesting good biocompatibility of the probiotic treatment.

In the second study (Peral et al. 2010), 14 patients (10 males, 4 females, aged 41–79 years) with chronic venous ulcers lasting more than three months and unresponsive to conventional treatments were treated with *L. plantarum* suspension once daily. The control group received standard wound care protocols. The study demonstrated significant bacterial load reduction, accelerated wound debridement, and improved granulation tissue formation, leading to complete healing in 43% of patients within 30 days. No significant adverse effects were observed, reinforcing the potential of probiotic therapy for chronic wounds [87].

Despite these positive clinical outcomes, the lack of a structured probiotic delivery system may have limited the full therapeutic potential of the treatment. In addition, both studies followed a non-randomized, open-label design, meaning that neither blinding nor randomization was implemented, which could introduce bias in outcome assessment.

### 3.3. Overview of the Functions of Probiotics, Synbiotics, and Postbiotics Identified in This Review

**Figure 3:**
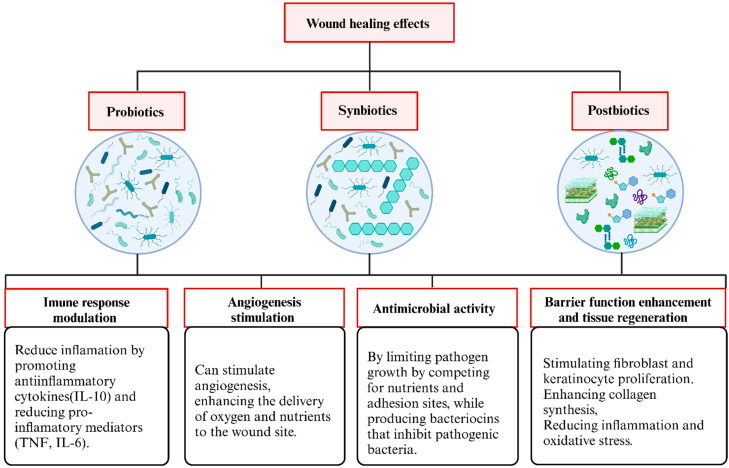
Summarized mechanisms of probiotics, synbiotics, and postbiotics in wound-healing environment.

Probiotic-based therapies offer a multifaceted approach to wound healing by modulating immune responses, enhancing antimicrobial defenses, and promoting tissue regeneration (Figure 3). One of the primary mechanisms identified in this review is immune response modulation, where probiotics stimulate the production of the anti-inflammatory cytokine IL-10 while simultaneously reducing pro-inflammatory mediators such as TNF-α and IL-6. This dual effect mitigates inflammation, creating an environment conducive to tissue repair and regeneration. Additionally, probiotics stimulate angiogenesis, improving oxygen and nutrient delivery to the wound site, supporting vascular regeneration, and accelerating wound closure while minimizing hypoxia-related complications.

Another crucial function of probiotics, synbiotics, and postbiotics is their antimicrobial activity, which helps limit pathogen growth by competing for nutrients and adhesion sites while producing bacteriocins that directly inhibit pathogenic bacteria. This mechanism maintains a balanced wound microbiota, reduces infection risk, and supports natural healing. Furthermore, barrier function enhancement and tissue regeneration occur by stimulating fibroblast and keratinocyte activity, increasing collagen synthesis, and promoting epidermal repair. Probiotics further accelerate tissue regeneration and strengthen the skin barrier by reducing oxidative stress and inflammation.

## 4. Expert Opinion and Considerations for Probiotic-, Synbiotic-, and Postbiotic-Based Wound Therapies

Using probiotics in wound healing represents a promising alternative to conventional antimicrobial treatments, particularly in infected wounds, where maintaining a balanced skin microbiome is essential for tissue regeneration. Among probiotic strains, *L. plantarum* stands out due to its robust antimicrobial effects against common wound pathogens, synthesis of bioactive metabolites including bacteriocins and organic acids, its ability to modulate inflammatory cytokines and promote angiogenesis, and its exceptional adaptability to various environmental settings. Together, these attributes likely explain its frequent selection and demonstrated effectiveness in topical wound healing research.

The development of living bacterial hydrogels and other advanced probiotic-based formulations could provide a biocompatible, antibiotic-free strategy for managing chronic and infected wounds. However, several challenges must be addressed before these therapies can be widely adopted in clinical practice. Gels are the most commonly used delivery system for probiotics, synbiotics, and postbiotics due to their hydrating properties, controlled release, and biocompatibility, creating a favorable environment for microorganisms and wound healing. Their ability to maintain moisture, promote epithelialization and collagen deposition, and reduce inflammation enhance tissue regeneration. Combining gels with wound dressings is essential to protect the wound, optimize healing conditions, and restore barrier function [24,30,35,39,41,42,50,100].

Despite these promising results, several challenges must be addressed before these therapies can be widely adopted in clinical practice. Key limitations in current research include the lack of direct comparisons with standard treatments, as many studies have not assessed probiotic-based therapies against conventional antibiotics, silver sulfadiazine, or growth factors. Another limitation is the absence of long-term stability data, raising concerns about probiotic viability and shelf life in formulated products. Additionally, while reductions in myeloperoxidase (MPO) activity suggest an anti-inflammatory effect, the specific molecular pathways involved, such as IL-10 or TNF-α modulation, remain unexplored. Moreover, this study represents an initial effort to gather evidence on the topic and is primarily descriptive/narrative.

The observed lack of transparency in randomization and allocation concealment in many animal studies can be attributed to the historical absence of standardized reporting practices in preclinical research, along with limited enforcement of methodological rigor during the design and publication phases. To enhance the quality and reproducibility of animal studies, it is essential to promote compliance with guidelines such as ARRIVE (Animal Research: Reporting of In Vivo Experiments) [67] and to encourage the preregistration of experimental protocols, which ensures improved documentation of randomization, blinding, and allocation processes.

The limited number of clinical trials identified highlights a gap between preclinical research and clinical application. This scarcity may reflect challenges such as the regulatory complexity of microbiome-based therapies, variability in formulation and strain selection, and the inherent difficulties in designing standardized clinical protocols for wound healing. Future research should focus on optimizing formulation stability, dosage standardization, and clinical validation to establish probiotic-based therapies as effective alternatives for wound management. Incorporating probiotics into optimized dermatological formulations, such as hydrogels, films, or microneedle patches, could enhance bacterial viability, ensure controlled release, and improve adhesion to the wound bed, creating a more favorable microenvironment for healing. More extensive clinical studies are needed to assess their efficacy, particularly in skin graft integration and long-term wound healing outcomes. Exploring synergistic combinations with other bioactive compounds may enhance therapeutic benefits, providing a scientifically robust and clinically viable alternative to conventional antimicrobial treatments.

Notably, no studies evaluating formulations containing only prebiotics for topical wound healing were identified. This absence may reflect the fact that prebiotics act primarily by promoting the growth and activity of beneficial microorganisms, such as probiotics, rather than exerting direct therapeutic effects. Consequently, their effectiveness depends on the presence of a viable and metabolically active microbiota, as in synbiotic formulations. The development and evaluation of prebiotic-only formulations thus represent a promising area for future research, particularly considering the potential for modulating resident skin microbiota without introducing live microorganisms.

Despite promising experimental results, the industrial-scale production of probiotic formulations—particularly those requiring high concentrations (≥10^10^ CFU/g)—faces important technical and economic challenges. Achieving these levels depends on the precise optimization of fermentation processes and nutrient media to ensure high yields while controlling costs [109,110]. Furthermore, maintaining microbial viability throughout processing and storage remains a critical bottleneck. Approaches such as microencapsulation or overfilling are often employed to preserve viability, but they can significantly increase production costs and limit scalability. Therefore, although technically feasible, the widespread therapeutic use of high-dose probiotic products will require overcoming economic and technological hurdles to ensure clinical effectiveness, manufacturing efficiency, and market accessibility.

## 5. Materials and Methods

The study was conducted following the guidelines for scoping review from the Joanna Briggs Institute (JBI), with data reported according to the PRISMA for Scoping Reviews (PRISMA-ScR) [111,112]. The protocol of the study is available at OSF https://doi.org/10.17605/OSF.IO/78UBA.

### 5.1. Search Strategy and Eligibility Criteria

The searches were conducted in the PubMed, Scopus, and Web of Science databases using terms related to cutaneous formulations, beneficial microbes and their derivatives, and skin disorders. Their respective synonyms were combined with Boolean operators such as “OR” and “AND” (Supplementary Table S3).

No timeframe or language limits were applied during the search. All studies retrieved from different databases were imported into Rayyan (Rayyan Systems, Inc., Doha, Qatar, free version), where duplicates were removed [113]. The eligibility criteria were based upon the PCC (Population, Concept, and Context) acronym, which was used to formulate the guide question: “What is the role of the formulations containing probiotics, prebiotics, synbiotics, and postbiotics for wound healing?”. The population was composed of topical formulations intended for cutaneous application. The concept explored included the use of probiotics, prebiotics, synbiotics, and postbiotics. The context of the application was wound healing.

Research articles focused on the development of formulations containing probiotics, prebiotics, synbiotics, or post-biotics, intended for the treatment of wounds, were selected. Only studies involving cutaneous formulations that evaluated in vitro, ex vivo, or in vivo performance were considered. Review articles, editorials, books, and conference abstracts were excluded. Studies on kefir, yogurt, and other dairy-derived probiotics were also excluded.

### 5.2. Study Selection

During the screening phase, two investigators independently assessed the titles and abstracts of the retrieved articles. Studies deemed relevant for the research were then evaluated in full according to the eligibility criteria by both reviewers. Any discrepancies between the reviewers’ assessments were resolved by a third author. The results of the identification, selection, and inclusion were reported in a flowchart.

### 5.3. Data Extraction and Synthesis

Studies included in this review had their metadata extracted in Microsoft Office Excel^®^ spreadsheets, including the following: (i) Publication data: title of articles, authors, year of publication, and location (country); (ii) Study details: Type of study, objectives, pharmaceutical form, production method, results of physicochemical characterization, tests of the formulations, and main outcomes. Again, two authors independently performed this task, with the participation of a third reviewer whenever necessary.

Qualitative data were first organized into categories based on predefined variables such as formulation type, biotic type, experimental model, and reported outcomes to perform a descriptive statistical analysis. We constructed an evidence gap map using Sankey plots to visualize the distribution and interconnections between these variables.

Sankey diagrams were chosen due to their capacity to represent flows and relationships between multiple categories, highlighting the volume and direction of connections. This type of visualization allows for an intuitive understanding of how evidence is distributed across different combinations of variables, for example, which formulations have been tested in which models and for what outcomes. The use of Sankey plots also enables the identification of clusters where evidence is concentrated and gaps where certain combinations remain unexplored.

Data analysis and the Sankey plots were created using Microsoft^®^ Excel for initial data organization and custom Python version 3.11.4 scripts employing the Plotly version 5.18.0 library for dynamic visualization (Supplementary Table S4). This approach provided a comprehensive and interactive way to synthesize large volumes of qualitative data, facilitating the identification of trends and research gaps in cutaneous formulations containing probiotics for wound healing.

### 5.4. Quality Assessment

The SYRCLE Risk of Bias Tool for animal studies was used to evaluate the quality of the included studies [114]. This tool assesses six key domains: selection bias, performance bias, detection bias, attrition bias, reporting bias, and other potential biases. The tool includes specific signaling questions to enhance clarity, assessment accuracy, and transparency. Each domain is classified as Yes, No, or Unclear, indicating a low risk of bias, a high risk of bias, or insufficient information to determine the risk, respectively.

## 6. Limitations

This review presents a comprehensive analysis of the available evidence on the use of probiotic, synbiotic, and postbiotic formulations in wound healing. Nevertheless, some limitations should be considered. The searches were conducted using only three scientific databases, without the use of manual search strategies, which may have led to the exclusion of relevant studies not retrieved by the applied terms. Finally, the inclusion of English language publications only may have reduced the comprehensiveness of the findings.

## 7. Conclusions

This study explored the therapeutic potential of formulations containing probiotics, synbiotics, and postbiotics for wound healing, highlighting their effect in modulating the immune response, controlling infections, and promoting tissue regeneration.

Animal studies have shown that different delivery systems, such as hydrogels, ointments, films, and powder-based microparticles, can optimize the stability and viability of beneficial microorganisms, providing prolonged and effective therapeutic effects. However, the risk of bias assessment highlighted methodological gaps, particularly in randomization, blinding, and animal allocation, emphasizing the need for greater experimental rigor to strengthen the scientific evidence.

Advances in biomaterial engineering and the development of delivery devices for beneficial microorganisms, such as adhesive patches and wound dressings, can further enhance the use of probiotics, synbiotics, and postbiotics in wound healing, fostering a balanced microenvironment and accelerating the regenerative process. However, to enable their integration into clinical practice, further robust clinical trials are required to confirm their efficacy and safety on a large scale, establishing these formulations as an innovative, biocompatible, and effective strategy for treating chronic and infected wounds.

## Figures and Tables

**Table 1:** Included studies on probiotic (live microorganisms) topical forms and skin disease applications.

Author/Year	Formulation	Probiotic Strain	In Vitro Activity	Type of Wound; In Vivo Model	Main Outcomes
Horikawa Y. (1986) [27]	Ointment	*Lactobacillus casei*	Not found	Burned skin; mice	The *L. casei* ointment improved healing of thermal injury wounds, eliminated bacteria, and enhanced collagen formation in mice. Its effects were superior to standard treatments, including the ointments Eksalb and Azunol and the creams mafenide acetate and silver sulfadiazine.
Brachkova, MI. et al. (2011) [28]	Film	*Lactobacillus plantarum*	Not found	Burned skin; Wistar rats	Films incorporating *L. plantarum* at cell concentrations of 10^8^ CFU/mL caused a 5–6 log10 reduction in *P. aeruginosa* in the model burn wounds. L. plantarum immobilized in freeze-dried calcium alginate films remained viable during six months of storage at 4 °C.
Jones M. et al. (2012) [29]	Adhesive patches	*Lactobacillus fermentum*	Antibacterial activity; *Trichophyton rubrum*, *Trichophyton mentagrophytes*, *E. coli*, *S. aureus* MRSA, *P. aeruginosa*, *Acinetobacter baumannii*	Excisional wound; New Zealand white rabbits	The study demonstrated the efficacy and safety of a probiotic patch containing lyophilized alginate microbeads with *L. fermentum* 7230, capable of producing gNO, for healing ischemic and infected wounds.
Oryan, A. et al. (2018) [30]	Scaffolds	*Saccharomyces cerevisiae*	Not found	Burned skin; Sprague Dawley rats	The CH-S biological dressing combined with the probiotic microorganism *S. cerevisiae* significantly increased collagen content and improved the biomechanical properties of healing burned wounds in rats.
Khan MA. et al. (2019) [31]	Scaffolds	*Enterococcus mundtii*	*S. aureus*	Burned skin; BALB/c mice	A comparative wound closure, histopathology, and wound microbial evaluation demonstrated that the bioscaffolds accelerate epithelialization, collagen deposition, and hair follicle formation, inhibit harmful bacteria, and provide interference benefits.
Khodaii Z. et al. (2019) [32]	Ointment	*Lactobacillus reuteri*	Not found	Excisional wound; Sprague Dawley rats	The probiotic ointment was effective for wound healing, including reducing inflammation, increasing collagen synthesis, decreasing lipid peroxidation and the activity of the MPO, speeding epithelialization, and increasing the percentage of wound contraction.
Rasheed, HT. et al. (2020) [33]	Emulgel	*Lactobacillus acidophilus*	Antibacterial activity: *P. aeruginosa*, *S. aureus*, and *Staphylococcus epidermidis*	Excisional wound; Albino mice	In vivo, *L. acidophilus* HT1 biomass effectively treated wounds infected with various bacterial pathogens within seven days, outperforming the control groups.
Ben David N. et al. (2021) [34]	Microparticles	*Bacillus subtilis*	Antibacterial activity; MRSA, *S. aureus*. Cytotoxicity; NIH 3T3 fibroblast.	Excisional wound; C57BL mice	*B. subtilis* in PVA microparticles showed strong antibacterial activity against MRSA and *S. aureus*. In in vivo experiments, both *B. subtilis* and empty PVA microparticles reduced healing time, with *B. subtilis* microparticles being more effective in the first week. No skin irritation, infection, or adverse effects were observed during the 15-day postoperative period.
Dubey AK. et al. (2021) [20]	Gel	*Lactiplantibacillus plantarum*	Human lung carcinoma	Excisional wound; BALB/c mice	Topical application of *Lp2621* to infected and uninfected wounds promoted rapid healing by enhancing angiogenesis, fibroblast proliferation, re-epithelialization, and recruitment of PMNLs.
Ming Z. et al. (2021) [35]	Hydrogel microspheres	*Lactobacillus reuteri*	Antibacterial activity; *E. coli*, *S. aureus*, and *Salmonella* spp. Cytocompatibility; Mouse fibroblasts L929	Excisional wound; BALB/c mice	This hydrogel, containing live bacteria, significantly reduced bacterial growth in infected skin, exhibited anti-inflammatory properties and effectively promoted wound healing and tissue regeneration.
Tsai, WH. et al. H. et al. (2021) [36]	Gel	*L. plantarum* GMNL-6, *Lacticaseibacillus paracasei* GMNL-653	Skin wound repair; Human foreskin fibroblasts	Excisional wound; BALB/c mice	Gels containing heat-killed GMNL-6 or GMNL-653 applied to experimental wounds on mouse tails promoted healing. Lipoteichoic acid provided anti-fibrogenic benefits similar to the heat-killed bacteria in the TGF-β-stimulated Hs68 fibroblast cell model.
Kazemi A. et al. (2022) [37]	Ointment	*Lactobacillus plantarum*, *Lactobacillus casei*	Antibacterial activity; *P. aeruginosa*. Cell viability; Bone marrow MSCs	Excisional wound; BALB/c mice	Probiotic metabolites and MSCs independently promote wound healing and, when administered together, exhibit a synergistic effect, leading to faster wound area reduction.
Kim, JS et al. (2022) [38]	Hydrogel, wound dressing	*Lactobacillus plantarum*	Not found	Excisional wound; Sprague Dawley rats	The guar-gum-based dual-layer wound dressing with *L. plantarum* demonstrated superior swelling capacity, mechanical properties, and promoted rapid wound recovery with complete re-epithelialization.
Mei L. et al. (2022) [39]	Hydrogel	*Lactobacillus rhamnosus*	Antibacterial activity; *P. aeruginosa*. Cytotoxicity assay; Mouse fibroblasts L929	Infected wound healing; Sprague Dawley rats	Hydrogel significantly suppressed bacteria-induced infection, increased the formation of re-epithelialization and collagen, and promoted wound healing, comparable to the commercial Prontosan gel.
Sousa MADS. et al. (2023) [24]	Gel	*Lactiplantibacillus plantarum*, *Lacticaseibacillus rhamnosus*, *Limosilactobacillus fermentum*,	Antibacterial activity; *S. aureus*, *Klebisiella pneumoniae*, *Enterococcus faecalis*, *P. aeruginosa*. Wound infection using porcine skin (ex vivo)	Not found	*Lactobacilli strains* incorporated into hydroxyethyl cellulose-based gels (Natrosol) showed antimicrobial effects. In the ex vivo assay using porcine skin, the LP-G18-A11 gel (5%) significantly reduced the skin loads of *S. aureus* and *P. aeruginosa* after 24 h. In contrast, only *P. aeruginosa* was reduced after 72 h.
Zhou C. et al. (2023) [19]	Hydrogel	*Lactobacillus reuteri*	Cytotoxicity; Mouse fibroblasts L929. Angiogenesis. Human umbilical vein endothelial	Excisional wound; BALB/c mice	The Gel/L@FeTA hydrogels presented a better performance than the Gel/L in inflammatory regulation, angiogenesis, and tissue regeneration both in in vitro and in vivo models in the presence of antibiotics.
Hua, C. et al. (2024) [40]	Microgels	*Lactobacillus fermentum*	Antibacterial activity; *P. aeruginosa*; Mouse fibroblasts L929 cells, Human umbilical venous endothelial cells (HUVECs) Angiogenesis.	Excisional wound; Sprague Dawley rats	The microgel system incorporating *Lactobacillus fermentum* and deferoxamine effectively managed multidrug-resistant *P. aeruginosa* and promoted wound healing. The system showed good biocompatibility and hemocompatibility.
Lu, Y. (2021) [41]	Hydrogel	*Lactococcus lactis*	Human umbilical vein endothelial (HUVECs), bone marrow-derived macrophages, *S. aureus*	Diabetic wound	This study introduces a thermoresponsive hydrogel with living *Lactococcus* and heparin-poloxamer to bioengineer the wound microenvironment and promote angiogenesis. The system enhances VEGF production, endothelial cell activity, and macrophage anti-inflammatory shifts, facilitating diabetic wound healing while minimizing systemic toxicity risks.
Yang, L. et al. (2020) [42]	Hydrogel	*Lactobacillus plantarum*	Antibacterial activity; *S. aureus*, *P. aeruginosa*, *E coli*. Cytotoxicity; Mouse fibroblasts L929.	Excisional wound; Kunming mice	The hydrogel with *Lactobacillus plantarum* exhibited potent antibacterial activity, excellent biocompatibility, and promoted L929 cell proliferation. In a full-thickness skin defect model, it accelerated wound healing by maintaining moisture, enhancing VEGF expression, reducing inflammation, boosting collagen deposition, and minimizing scarring.
Huang, B. et al. (2024) [43]	Nanofiber films	*Lactobacillus paracasei*	Antibacterial activity; *E. coli*, *S. aureus*	Infected wound healing; SD rats	*L. paracasei* biofilms demonstrated superior antibacterial activity against pathogenic bacteria, including S. aureus, given their ability to activate M2 macrophages, which are key participants in the immune response and tissue repair processes.
Jin, Y. et al. (2024) [44]	Microneedles patch	*Lactobacillus reuteri*	Antibacterial activity; *S. aureus*, *P. aeruginosa*, and *E. coli*. NIH-3T3 cells, Human umbilical venous endothelial cells (HUVECs)	Excisional wound; SPF BALB/c female mice,	In a mouse model of *Staphylococcus aureus*-infected wounds, a single administration of the microneedle patch exhibited superior antimicrobial efficiency and wound healing performance compared with control groups.

**Table 2:** Included studies on components of synbiotic (live microorganisms with prebiotics) and wound-healing applications.

Author/Year	Formulation	Probiotic Strain	Prebiotic	In Vitro Activity	Type of Wound; In Vivo Model	Main Outcomes
Cerchiara et al. (2020) [45]	Freeze-dried dressings	*Lactobacillus plantarum*	Vitamin E	Biocompatibility in human fibroblast; Antibacterial activity against *S. aureus* and *P. aeruginosa*	Not found	These formulations were not toxic to human fibroblast cells and assured a sustained release of Vitamin E, preserving its antioxidant property, and showing good antibacterial activity against *S. aureus* and *P. aeruginosa*.
Kizhakkekalam et al. (2022) [46]	Gel	*Bacillus amyloliquefaciens*	Macroalgal polysaccharide	Cell migration studies on L929 cell lines; Antibacterial activity against *P. aeruginosa* MDR, *S. pyogenes*, *E. coli*, *S. aureus* MRSA, *K. pneumoniae*	Not found	The topical formulation containing the organic extract of marine synbiotic *B. amyloliquefaciens* MTCC 12716 stimulated epithelial wound healing and improved wound closure. Promising antibacterial properties against clinical wound isolates were also reported
Guan et al. (2023) [47]	Spray/hydrogel film	*Lactobacillus casei*	Flavones	Biosecurity using fibroblast NIH-3T3 cells and red blood cells, and chorioallantoic membrane (CAM) test; Antibacterial assessment against MRSA and *E. coli*	Infected burn wound; Wister rats	The instant protection spray formed a protective barrier for burns within 30 s, sterilizing 100% of MRSA in vitro and 96.14% in vivo.
Farahani et al. (2023) [22]	Powder particles	*Lactiplantibacillus plantarum*	FOS	Not found	Infected burn wound; Wistar rats	Chitosan-alginate particles showed antibacterial activity and accelerated wound healing. FOS * enhanced *L. plantarum* stability and survival.
Sandhu et al. (2023) [48]	Film	*Lactobacillus plantarum* UBLP-40	Curcumin	Antimicrobial activity against *S. aureus*	Excisional wound; Lacca mice	Curcumin-loaded SLNs with probiotics boosted antimicrobial effects against *S. aureus* by 560%, accelerated wound closure, reduced bioburden and inflammation, and enhanced healing through growth factors and antioxidants.
Yang et al. (2024) [49]	Hydrogel	*Lactobacillus plantarum*	FOS	Cytotoxicity in fibroblasts NIH/3T3 and human umbilical venous endothelial cells; Cell migration assay in fibroblasts NIH/3T3; Antibacterial activity against *S. aureus* and *P. aeruginosa*	Diabetic infectious wounds; Sprague Dawley rats	A living microecological hydrogel containing *L. plantarum* and FOS * (LP/FOS@Gel) remodeled dysregulated skin microbiota, promoted the proliferation of beneficial bacteria, eliminated pathogenic colonization, and modulated immune responses.
Xu, H. et al. (2024) [50]	Hydrogel	*Lactobacillus paracasei*, *Bacillus velezensis*	Extracellular polysaccharide EPS-M76	Antibacterial activity; *E. coli*, *S. aureus*; L929 cells	Excisional wound; SD rats	Live probiotic hydrogels reduced the incidence of inflammation during wound healing by promoting angiogenesis and increasing collagen deposition.

* FOS: Fructooligosaccharide.

**Table 3:** Included studies on postbiotic (non-viable components or metabolites) and wound healing applications.

Author/Year	Formulation	Probiotic Strain	Postbiotic	In Vitro Activity	Type of Wound; In Vivo Model	Main Outcomes
Zouari et al. (2016) [51]	Gel	*Bacillus subtilis* SPB1	Crude lipopeptide biosurfactant from cell-free supernatant	Not found	Excisional wound; Wistar rats	The gel containing biosurfactant accelerated wound healing, with lipopeptides showing strong antioxidant, antimicrobial, and antifungal properties.
Kalenova et al. (2017) [52]	Ointment	*Bacillus* sp.	Cell-free metabolites	Not found	Excisional wound; BALB/c mice	*Bacillus* sp. metabolites promoted 30% faster epithelialization, enhanced immunity, reduced scarring, and supported hair recovery, outperforming Solcoseryl.
Moghadam, S.S. et al. (2020) [53]	Ointment	*Lactobacillus plantarum*	Cell-free supernatant	Not found	Burned skin; Wistar rats	Ointment containing the *L. plantarum* supernatant had a significantly smaller wound size than the imipenem group. Histological analysis revealed better skin repair in the probiotic cell pellet group.
Halper, J. et al. (2003) [54]	Gel	*Lactobacillus acidophilus*	Cell-free supernatant	Mouse embryonal kidney fibroblastic AKR-2B; Murine macrophage J774.A1; Porcine kidney LLC-PK1	Excisional wound; Swiss NIH mice; Sprague Dawley rats	The study demonstrates the potential of *Lactobacillus* strains (ATCC 4356 and 43121) as stimulators of the inflammatory stage of tissue repair, TNF-alpha production, and angiogenesis.
Sinha, A. et al. (2019) [55]	Gel	*Lactobacillus* (VITSAMJ1)	Cell-free supernatant	Antibacterial activity; *S. aureus*	Excisional wound; Wistar rats	Animals treated with the probiotic gel showed better wound healing compared to the control groups.
Ashoori, Y. et al. (2020) [18]	Nanogel	*Bacillus subtilis* sp. *natto*, *Lactobacillus fermentum*, *Lactobacillus reuteri*.	Cell-free supernatant	Not found	Excisional wound; Sprague- Dawley rats	*B. subtilis* sp. natto has a better wound healing efficacy, as demonstrated in pathology examination. Favorable effects of probiotic lysate nanogels, including the reasonable wound closing rate, good wound appearance, and good histological observation, were confirmed in vivo.
Golkar et al. (2021) [56]	Cream	*Bacillus subtilis* sp. *natto*, *Lactobacillus reuteri*, *Lactobacillus fermentum*	Cell-free supernatant	Not found	Excisional wound; Sprague Dawley rats	Postbiotic formulations accelerated wound healing. *B. subtilis* natto cold cream showed the best results
Jamaran et al. (2021) [26]	Film	*Lactobacillus reuteri*	Cell-free supernatant	Antibacterial activity against *P. aeruginosa*, *S. aureus*, and *E. coli*	Excisional wound; Wistar rats	The postbiotic/CS/PEG treatment accelerated wound healing, enhanced cytokine and chemokine expression, promoted immune cell activity, and improved collagen and elastin deposition, enhancing wound integrity.
Shokatayeva et al. (2021) [57]	Film	*Bacillus subtilis P-2*	Cell-free supernatant	Antibacterial activity against *E. coli*, *P. aeruginosa*, *S. aureus*, and *S. epidermidis*	Excisional wound; Mongrel rats	Postbiotic integrated into a biocomposite of bacterial cellulose and chitosan reduced wound healing time by 20% in animals, being also effective against Gram-positive and Gram-negative bacteria.
Bazjoo, A. et al. (2022) [21]	Film	*Bifidobacterium bifidum*	Cell-free supernatant	Not found	Excisional wound; Wistar rats	The biodegradable film based on chitosan and CFS of *B. bifidum* improved the wound healing process.
Nazari et al. (2024) [58]	Fiber/ Hydrogel (hybrid wound dressing)	*Lactobacillus plantarum*	Exopolysaccharide	Cell viability, proliferation, and attachment using human dermal fibroblast (HDF)	Not found	A fiber–hydrogel dressing using eggshell membrane enriched with postbiotic compounds (EPS) from *L. plantarum* (10 mg/mL) enhanced cell proliferation within five days.
Ekrami et al. (2024) [59]	Nanofibrous membrane	*Bacillus coagulans*	Lactosporin	Cell toxicity in L929 mouse fibroblast; Antibacterial activity against *M. luteus*, *E. coli*, *Pseudomonas*, *S. aureus*, *S. epidermis*, and *K. pneumoniae*	Excisional wound; Sprague Dawley male rats	The hyaluronic acid-based nanofibers loaded with Lactosporin demonstrated antimicrobial efficacy, which was favorable for the wound healing process.
Kuhn, T. et al. (2024) [60]	Hydrogel on membrane	*Lactobacillus casei*, *Lactobacillus plantarum*	Cell-free extracellular vesicles	Viability assay; Human immortal keratinocyte HaCaT; Monocyte-like THP-1; Peripheral blood mononuclear	Excisional wound; Mice	Hydrogels containing cell-free extracellular vesicles derived from *L. casei* and *L. plantarum* improved healing in an in vivo mouse full-thickness wound model.
Dubey, AK. et al. (2023) [61]	Gel	*Lactiplantibacillus plantarum*	Cell-free supernatant	Immune respond; Human leukemia monocyte cell line T-helper; Antibacterial activity; MRSA	Excisional wound; BALB/c mice	Lp2621, a probiotic cell-free supernatant (CFS), had potent antibacterial and antioxidant properties. It also exhibited in vitro biofilm inhibition and eradication activity and anti-MRSA activity.
Qi, F. et al. (2024) [62]	Microneedle patch	*Lactobacillus druckerii*	Extracellular vesicles	Antibacterial activity; *Staphylococcus aureus*, *Escherichia coli*; HaCaT; Murine fibroblasts	Excisional wound; Balb/c mice	Core–shell microneedle with sequential delivery of tannic acid–magnesium (TA-Mg) complexes and Lactobacillus druckerii extracellular vesicles (LDEVs). CSMN@TA-Mg/LDEV increased microbial diversity at wound sites.
Hausmann, C. et al. (2019) [63]	Emulsion	*Lactococcus lactis*	*Lactococcus lactis* lysate	Reconstructed human epidermis	A clinical trial was conducted with 21 women (aged 35–59 years) with Fitzpatrick skin type II–IV, and it was a randomized controlled double-blind trial.	*L. lactis* formulations enhance the skin barrier by increasing filaggrin and β-defensin-2 expression, reducing TEWL by 18%, and lowering permeability to caffeine. They also improve hydration and surface pH without causing irritation.

Note: CFS—Cell-free supernatant, EPS—Exopolysaccharide.

## Data Availability

No new data were created or analyzed in this study. Data sharing is not applicable.

## Supplementary Materials

The following supporting information can be downloaded at: https://www.mdpi.com/article/10.3390/ph18050704/s1, Tables S1–S4: Data extraction.
