# Supplementary material for: Next-Generation Wound Care: A Scoping Review on Probiotic, Prebiotic, Synbiotic, and Postbiotic Cutaneous Formulations
Source: Pharmaceuticals (Basel). 2025 May 9;18(5):704. doi: 10.3390/ph18050704 (PMC12114949; doi:10.3390/ph18050704)
Supplement: Supplementary file 1 [file pharmaceuticals-18-00704-s001.zip › pharmaceuticals-3596486-supplementary/Supplementary Table S3.pdf]

Table S3. Search strategy.

PubMed
#1 AND #2 AND #3<br><br>#1<br>(((((((((((skin[Title/Abstract]) OR (dermatitis[Title/Abstract])) OR (wound*[Title/Abstract])) OR (acne[Title/Abstract])) OR (psoriasis[Title/Abstract])) OR ("skin carcinogenesis"[Title/Abstract])) OR (rosacea[Title/Abstract])) OR (dyspigmentation[Title/Abstract])) OR (dandruff[Title/Abstract])) OR ("dry skin"[Title/Abstract])) OR (eczema[Title/Abstract])) OR (dysbiosis[Title/Abstract])) OR (aging[Title/Abstract])<br><br>#2<br>(((((((probiotic*[Title/Abstract]) OR (prebiotic*[Title/Abstract])) OR (synbiotic*[Title/Abstract])) OR (postbiotic*[Title/Abstract])) OR (lactobacillus[Title/Abstract])) OR (bifidobacterium[Title/Abstract])) OR (vitreoscilla[Title/Abstract])) OR (bacillus[Title/Abstract])<br><br>#3<br>(((((((((((microparticle*[Title/Abstract]) OR ("topical formulation"[Title/Abstract])) OR (cream[Title/Abstract])) OR (gel[Title/Abstract])) OR (film[Title/Abstract])) OR (pats[Title/Abstract])) OR (ointment*[Title/Abstract])) OR (lotion[Title/Abstract])) OR (lotion[Title/Abstract])) OR (emulsion*[Title/Abstract])) OR (patch[Title/Abstract])) OR (foam[Title/Abstract])) OR ("cutaneous formulation*"[Title/Abstract])
**Scopus**
#1 AND #2 AND #3<br><br>#1<br>(TITLE-ABS-KEY (skin) OR TITLE-ABS-KEY (dermatitis) OR TITLE-ABS-KEY (wound*) OR TITLE-ABS-KEY (acne) OR TITLE-ABS-KEY (psoriasis) OR TITLE-ABS-KEY ("skin carcinogenesis") OR TITLE-ABS-KEY (rosacea) OR TITLE-ABS-KEY (dyspigmentation) OR TITLE-ABS-KEY (dandruff) OR TITLE-ABS-KEY ("dry skin") OR TITLE-ABS-KEY (eczema) OR TITLE-ABS-KEY (dysbiosis) OR TITLE-ABS-KEY (aging))<br>#2<br>(TITLE-ABS-KEY (probiotic*) OR TITLE-ABS-KEY (prebiotic*) OR TITLE-ABS-KEY (synbiotic*) OR TITLE-ABS-KEY (postbiotic*) OR TITLE-ABS-KEY (lactobacillus) OR TITLE-ABS-KEY (bifidobacterium) OR TITLE-ABS-KEY (vitreoscilla) OR TITLE-ABS-KEY (bacillus))<br>#3<br>(TITLE-ABS-KEY (microparticle*) OR TITLE-ABS-KEY ("topical formulation") OR TITLE-ABS-KEY (cream) OR TITLE-ABS-KEY (gel) OR TITLE-ABS-KEY (film) OR TITLE-ABS-KEY (pats) OR TITLE-ABS-KEY (ointment*) OR TITLE-ABS-KEY (lotion) OR TITLE-ABS-KEY (emulsion*) OR TITLE-ABS-KEY (patch) OR TITLE-ABS-KEY (foam) OR TITLE-ABS-KEY ("cutaneous formulation*"))
**Web of Science**
#1 AND #2 AND #3<br><br>#1<br>(((((((((((TS=(skin)) OR TS=(dermatitis)) OR TS=(wound*)) OR TS=(acne)) OR TS=(psoriasis)) OR TS=("skin carcinogenesis")) OR TS=(rosacea)) OR TS=(dyspigmentation)) OR TS=(dandruff)) OR TS=("dry skin")) OR TS=(eczema)) OR TS=(dysbiosis)) OR TS=(aging)

#2
(((((((TS=(probiotic*)) OR TS=(prebiotic*)) OR TS=(synbiotic*)) OR TS=(postbiotic*)) OR TS=(lactobacillus)) OR TS=(bifidobacterium)) OR TS=(vitreoscilla)) OR TS=(bacillus)

#3
(((((((((((TS=(microparticle*)) OR TS=("topical formulation")) OR TS=(cream)) OR TS=(gel)) OR TS=(film)) OR TS=(pats)) OR TS=(ointment*)) OR TS=(lotion)) OR TS=(emulsion*)) OR TS=(patch)) OR TS=(foam)) OR TS=("cutaneous formulation*")
